# HDGF promotes gefitinib resistance by activating the PI3K/AKT and MEK/ERK signaling pathways in non-small cell lung cancer

**DOI:** 10.1038/s41420-023-01476-0

**Published:** 2023-06-10

**Authors:** Shuyan Han, Zhihua Tian, Huifang Tian, Haibo Han, Jun Zhao, Yanna Jiao, Chunli Wang, Huifeng Hao, Shan Wang, Jialei Fu, Dong Xue, Hong Sun, Pingping Li

**Affiliations:** 1 Department of Integration of Chinese and Western Medicine, Key laboratory of Carcinogenesis and Translational Research (Ministry of Education), Peking University Cancer Hospital & Institute, Beijing, 100142 China; 2 Central Laboratory, Key laboratory of Carcinogenesis and Translational Research (Ministry of Education), Peking University Cancer Hospital & Institute, Beijing, 100142 China; 3 The Tissue Bank, Key laboratory of Carcinogenesis and Translational Research (Ministry of Education), Peking University Cancer Hospital & Institute, Beijing, 100142 China; 4 Department of Thoracic Medical Oncology, Key laboratory of Carcinogenesis and Translational Research (Ministry of Education), Peking University Cancer Hospital & Institute, Beijing, 100142 China; 5 Department of Oncology, Infectious Disease Hospital of Heilongjiang Province, Harbin, 150030 China

**Keywords:** Non-small-cell lung cancer, Drug development, Predictive markers

## Abstract

Hepatoma-derived growth factor (HDGF) expression is associated with poor prognosis in non-small cell lung cancer (NSCLC); however, whether HDGF affects gefitinib resistance in NSCLC remains unknown. This study aimed to explore the role of HDGF in gefitinib resistance in NSCLC and to discover the underlying mechanisms. Stable HDGF knockout or overexpression cell lines were generated to perform experiments in vitro and in vivo. HDGF concentrations were determined using an ELISA kit. HDGF overexpression exacerbated the malignant phenotype of NSCLC cells, while HDGF knockdown exerted the opposite effects. Furthermore, PC-9 cells, which were initially gefitinib-sensitive, became resistant to gefitinib treatment after HDGF overexpression, whereas HDGF knockdown enhanced gefitinib sensitivity in H1975 cells, which were initially gefitinib-resistant. Higher levels of HDGF in plasma or tumor tissue also indicated gefitinib resistance. The effects of HDGF on promoting the gefitinib resistance were largely attenuated by MK2206 (Akt inhibitor) or U0126 (ERK inhibitor). Mechanistically, gefitinib treatment provoked HDGF expression and activated the Akt and ERK pathways, which were independent of EGFR phosphorylation. In summary, HDGF contributes to gefitinib resistance by activating the Akt and ERK signaling pathways. The higher HDGF levels may predict poor efficacy for TKI treatment, thus it has the potential to serve as a new target for overcoming tyrosine kinase inhibitor resistance in combating NSCLC.

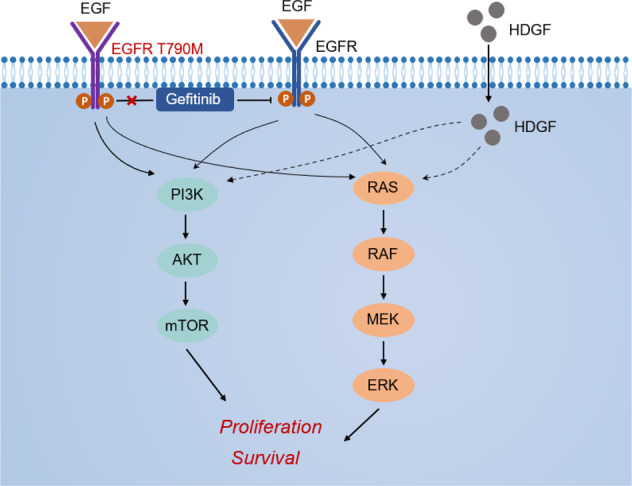

## Introduction

Lung cancer is the most common cause of cancer-related death worldwide. Non-small cell lung cancer (NSCLC) accounts for approximately 80% of all lung carcinoma cases. Patients with epidermal growth factor receptor (EGFR) mutations respond well to tyrosine kinase inhibitors (TKIs), but all responding patients eventually develop acquired resistance to TKIs [1]. The underlying mechanisms of TKI resistance include secondary resistance mutations of targeted genes, activation of bypass signaling pathways, dysregulation of downstream effectors, and phenotypic transformation [2]. T790M mutation in exon 20 of EGFR accounts for approximately 50% of resistant cases and is the predominant resistance mechanism of TKIs. However, approximately 19% of resistance mechanisms remain unknown [3].

Hepatoma-derived growth factor (HDGF) is a heparin-binding growth factor that was first identified from the conditioned medium of the Huh-7 hepatoma cell line [4], indicating that it can be secreted. HDGF has been reported to be involved in cancer cell growth, angiogenesis, anti-apoptosis, and tumor metastasis [4, 5]. In a range of tumor types, including NSCLC, ovarian cancer, oral cancer, gastric cancer and melanoma, HDGF is overexpressed, and its higher expression is strongly correlated with poor prognosis [6–11]. HDGF enhances vascular endothelial growth factor (VEGF)-dependent angiogenesis in NSCLC [12], and high serum HDGF levels predict bone metastasis and unfavorable prognosis in NSCLC [13]. In surgically resected stage I NSCLC, HDGF is regarded as a biomarker for molecular staging and prognosis and provides prediction for postoperative adjuvant chemotherapy [14]. Inhibition of HDGF suppressed tumor cell growth both in vitro and in vivo [15, 16]. Antibodies targeting HDGF could prevent NSCLC relapse after chemotherapy by suppressing cancer stem cells [17, 18]. Some microRNAs (miRNAs) inhibit NSCLC proliferation and invasion by targeting HDGF [19–21]. Thus, HDGF is a promising potential target for NSCLC treatment.

Our previous experiment demonstrated that *Marsdenia tenacissima* extract (MTE) could overcome resistance to gefitinib, a comprehensively used first-generation TKI, in NSCLC in vitro and in vivo [22, 23]. Meanwhile, MTE could disturb the interaction between tumor-associated macrophages and NSCLC cells by targeting HDGF [24]. Our preliminary study showed that MTE combined with gefitinib could decrease HDGF expression in resistant H1975 cells, as evidenced by 2D-gel electrophoresis coupled with high-performance liquid chromatography-tandem mass spectrometry (HPLC‒MS/MS) and Western blotting (Fig. S1). Studies have indicated that HDGF promotes chemotherapy resistance in colorectal cancer cells and tongue squamous cell carcinoma [25, 26], and it drives radioresistance in breast cancer [27]. However, the role of HDGF in gefitinib resistance is unclear. Although site-specific peptide reporters and targeted mass spectrometry indicated that HDGF-S165, a synthetic substrate peptide, may have a potential association with TKI resistance [28], there is no direct evidence associating HDGF levels and TKI efficacy. In this study, we demonstrated the correlation between HDGF and gefitinib resistance in vitro and in vivo and identified the complementary effect between HDGF and EGFR in NSCLC cells.

## Results

### Downregulation of HDGF may be involved in overcoming gefitinib resistance by MTE

Through 2D gel and LC‒MS/MS analyses, HDGF was identified as one of the differentially expressed proteins in H1975 cells treated with gefitinib or MTE alone or in combination (Fig. S1). Studies have indicated that HDGF expression is regarded as a prognostic factor in patients with early-stage NSCLC [6]; HDGF also promotes chemotherapy resistance in some tumors, including colorectal cancer, tongue squamous cell cancer, and breast cancer [25–27]. Therefore, we chose HDGF as the target protein for further study. Western blot results showed that HDGF was significantly downregulated after exposure to combined gefitinib and MTE treatment compared to each single treatment and the control group in H1975 cells (Fig. S1).

### HDGF regulated the malignant properties of NSCLC cells

**Fig. 1:**
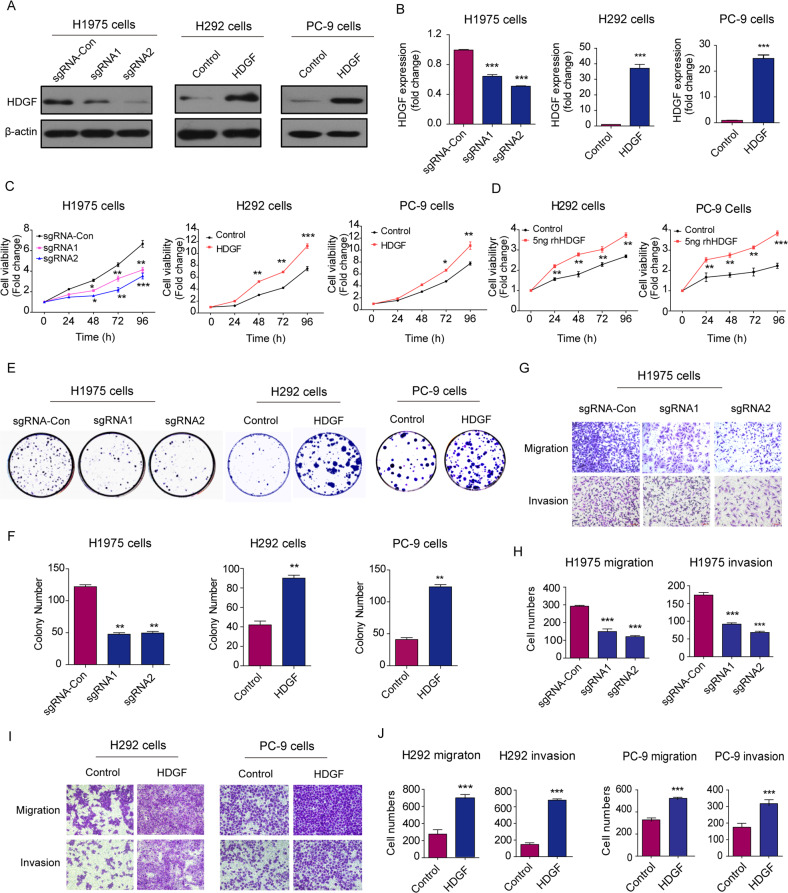
HDGF promoted the malignant phenotype of NSCLC cells. HDGF was knocked down by the CRISPR/Cas9 system in H1975 cells, while it was overexpressed in PC-9 and H292 cells by transfecting the plenti6-TR plasmid. HDGF expression was detected by Western blotting (**A**) and qRT‒PCR. **B** Proliferation of NSCLC cells with different levels of HDGF expression (**C**) or stimulated with 5 ng rhHDGF (**D**) was detected at 24, 48, 72 and 96 h. **E** Colony formation abilities of NSCLC cells with different HDGF expression levels. **F** Shows the analytical results of **E**. **G** The migration and invasion abilities of HDGF-knockdown H1975 cells were determined by Transwell assay. **H** Shows the analytical results of (**G**). **I** Migration and invasion abilities were determined in HDGF-overexpressing PC-9 and H292 cells. **J** shows the analytical results of (**I**). Data are from one representative experiment performed in triplicate. ^*^P < 0.05, ^**^P < 0.01, ^***^P < 0.005 vs. control.

To further understand the function of HDGF in NSCLC, we detected its expression in seven NSCLC cell lines with different responses to gefitinib. The gefitinib-sensitive PC-9 and H292 cells had relatively lower HDGF expression, while H1975 cells expressed very high levels of HDGF (Fig. S1). HDGF was knocked down by the CRISPR/Cas9 system in H1975 cells, while stable HDGF overexpression was established by transfecting the HDGF plenti6-TR plasmid into PC-9 and H292 cells. HDGF overexpression or knockdown was validated by Western blotting and qRT‒PCR (Figs. 1A, 1B).

To understand the influence of HDGF levels on the biological characteristics of NSCLC cells, we measured cell phenotypes after HDGF knockdown or overexpression. As shown in Fig. 1, HDGF sgRNA1 and sgRNA2 significantly inhibited H1975 the cell proliferation (Fig. 1C), colony formation (Fig. 1E, F), and migration and invasion (Fig. 1G, H) abilities (*P* < 0.01, *P* < 0.001 vs. control), indicating that HDGF knockdown reduced the malignant properties of NSCLC cells. In contrast, H292 and PC-9 cell proliferation was promoted by HDGF overexpression (Fig. 1C) or stimulated with rhHDGF (Fig. 1D). Meanwhile, HDGF overexpression amplified the colony formation (Fig. 1E, F) and migration and invasion (Fig. 1I, J) abilities of H292 and PC-9 cells (*P* < 0.01, *P* < 0.001 vs. control). Therefore, the above data demonstrated that HDGF promoted the malignant phenotype of NSCLC cells.

### HDGF expression levels affected NSCLC tumor growth in vivo

**Fig. 2:**
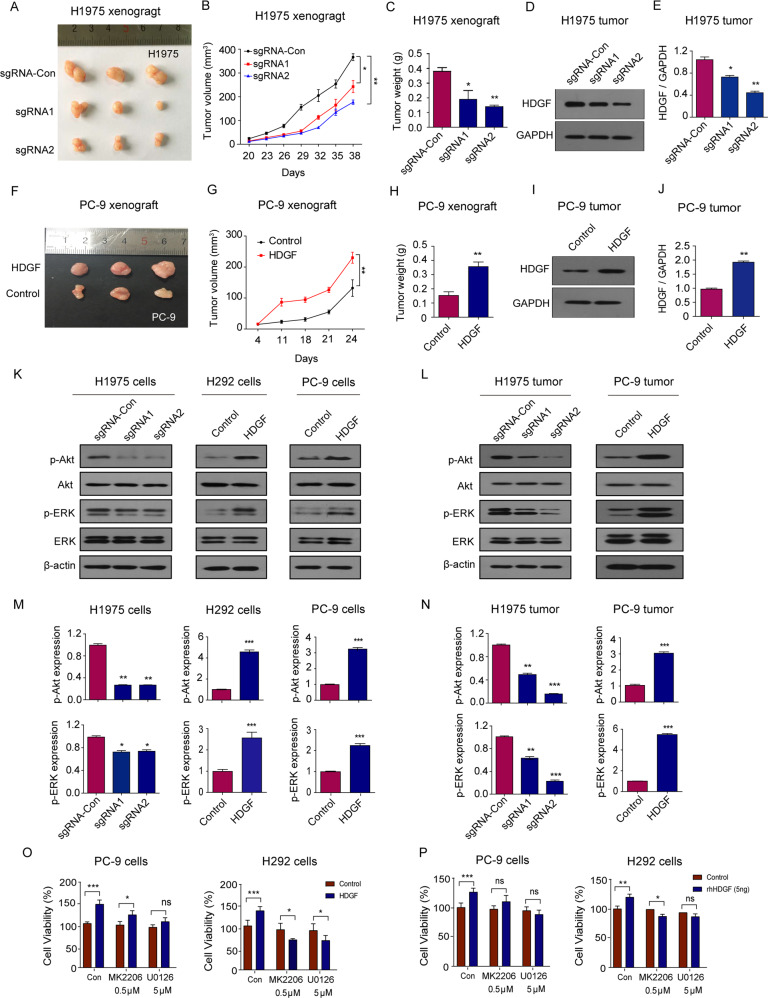
HDGF regulated NSCLC tumor growth and the gefitinib resistance-associated molecules p-Akt and p-ERK. **A** Representative images of H1975 xenograft tumors with HDGF knockdown. **B**, **C** Show H1975 xenograft tumor volume and tumor weight. **D** HDGF protein expression in H1975 mouse tumor tissues. **E** Shows the analytical results of (**D**). **F** Representative images of PC-9 xenograft tumors in nude mice with HDGF overexpression. **G**, **H** Show PC-9 xenograft tumor volume and tumor weight. **I** HDGF protein expression in PC-9 mouse tumor tissues. **J** Shows the analytical results of I. The expression of p-Akt and p-ERK in H1975 and PC-9 cells (**K**) or mouse tumor tissues (**L**) with HDGF knockdown or overexpression. **M,** **N** Show the analytical results of (**K**, **L**). **O**, **P** HDGF overexpression or rhHDGF**-**induced H292 and PC-9 cell growth was abrogated or reversed by the Akt inhibitor MK2206 or the ERK1/2 inhibitor U0126. Data are from one representative experiment performed in triplicate. ^*^P < 0.05, ^**^P < 0.01, ^***^P < 0.005 vs. control.

We determined the association between HDGF and tumor growth in xenograft mice. As shown in Fig. 2, compared with the control group, tumor growth was significantly decreased by both HDGF sgRNAs (Fig. 2A–C), and the inhibitory effect was more obvious in the sgRNA2 group, indicating that HDGF knockdown restricted H1975 tumor development. Meanwhile, Western blotting confirmed that HDGF expression was inhibited by both sgRNAs in H1975 tumor tissues (Fig. 2D, E). Conversely, HDGF overexpression markedly augmented PC-9 tumor volume and weight when compared with the control group (Fig. 2F–H). Western blot assays also confirmed higher levels of HDGF in PC-9 tumor tissues (Fig. 2I, J). The above results indicated that HDGF promoted tumor growth but that HDGF knockdown could restrict this process.

### HDGF regulated EGFR downstream p-Akt and p-ERK

Studies have demonstrated that the EGFR downstream molecules p-Akt and p-ERK are abnormally activated when NSCLC exerts resistance to gefitinib [29]. In our experiment, alterations in these two molecules were determined in NSCLC cells and mouse tumor tissues. Compared with the control group, HDGF knockdown resulted in dramatically decreased p-Akt and p-ERK expression in H1975 cells, and sgRNA2 showed a more potent inhibitory effect. In contrast, HDGF overexpression augmented p-Akt and p-ERK levels in H292 and PC-9 cells (Fig. 2K, M). In mouse tumor tissues, the changes in p-Akt and p-ERK were consistent with those in the cell lines (Fig. 2L, N). In the presence of the Akt inhibitor MK2206 or the ERK inhibitor U0126, the enhanced growth induced by HDGF overexpression or rhHDGF in H292 and PC-9 cells was abrogated or reversed (Fig. 2O, P).

### HDGF is associated with gefitinib efficacy in vitro

**Fig. 3:**
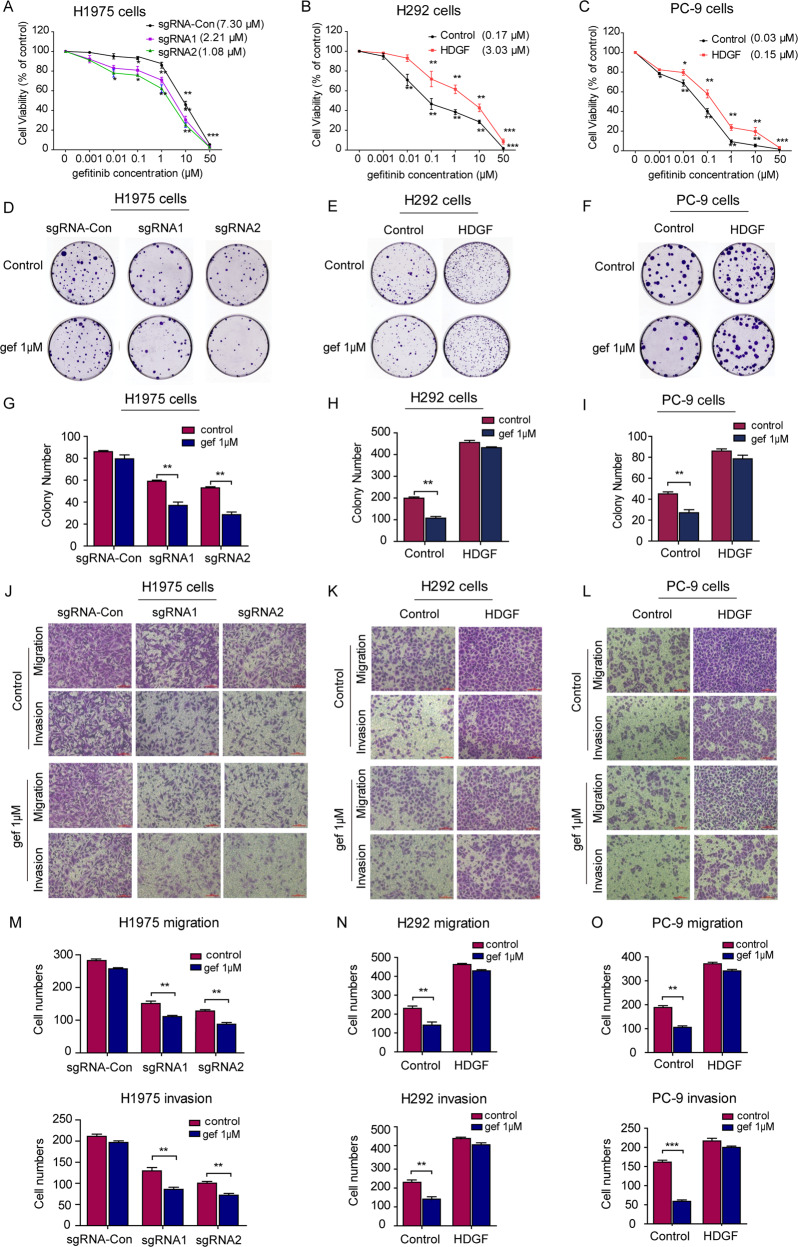
HDGF knockdown or overexpression influenced gefitinib efficacy in NSCLC cell lines. **A**–**C** H1975, PC-9, and H292 cells were treated with 0.001 ~ 50 μM gefitinib for 72 h. **D**–**F** NSCLC cell colony formation ability was detected after gefitinib treatment. **G**–**I** Show the analytical results of **D**–**F**. **J**–**L** NSCLC cell migration and invasion capacities were detected after gefitinib treatment for 24 h. **M**–**O** Show the analytical results of J-L. Data are from one representative experiment performed in triplicate. ^*^P < 0.05, ^**^P < 0.01, ^***^P < 0.005 vs. control.

After understanding the role of HDGF in NSCLC, we further studied the association between HDGF expression and gefitinib efficacy. As shown in Fig. 3A, HDGF knockdown by sgRNAs in H1975 cells responded well to gefitinib, with IC50 values of 2.21 μM and 1.08 μM, respectively, compared to 7.30 μM in the sgRNA control group. In contrast, HDGF augmented gefitinib IC50 values by 17- and 20-fold compared with gefitinib-sensitive parental H292 and PC-9 cells (Fig. 3B, C). Gefitinib treatment markedly suppressed the colony formation and migration and invasion abilities in HDGF-silenced H1975 cells but only exerted minor influences in the control group (Fig. 3D, G, J, M). Conversely, gefitinib treatment reduced the colony formation (Fig. 3E, F, H, I) and migration and invasion capacities (Fig. 3K, L, N, O) of H292 and PC-9 cells, but these inhibitory effects were abrogated after HDGF overexpression. The above results suggested that higher HDGF expression may be associated with gefitinib resistance.

### HDGF is associated with gefitinib efficacy in vivo

**Fig. 4:**
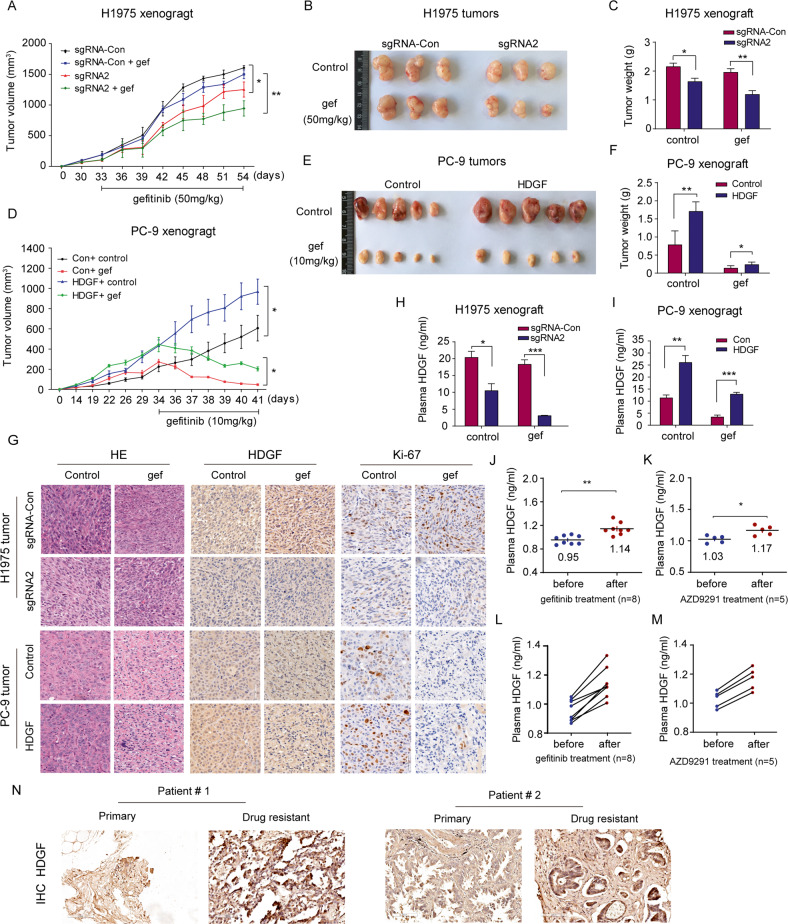
HDGF is associated with gefitinib efficacy in NSCLC in vivo. NSCLC cells were subcutaneously inoculated in nude mice, which were then administered gefitinib for the indicated consecutive days after tumor formation. Tumor volume, representative images of tumors, and tumor weight of HDGF-knockdown H1975 xenografts treated with 50 mg/kg gefitinib (**A**–**C**) or HDGF-overexpressing PC-9 xenografts treated with 10 mg/kg gefitinib (**D**–**F**). **G** HDGF and Ki-67 expression levels in H1975 and PC-9 tumor tissues (20×). **H, I** Plasma HDGF concentrations in H1975 and PC-9 xenografts. **J**, **K** Plasma HDGF concentrations in NSCLC patients before receiving gefitinib or AZD9291 (a third-generation TKI) and after drug resistance occurred. **L**, **M** Plasma HDGF levels in each patient before and after receiving gefitinib or AZD9291. **N** HDGF expression in biopsy tissue samples measured by IHC before TKI treatment and after disease progression. Patient #1 received icotinib treatment and patient #2 administered AZD9291. ^*^P < 0.05, ^**^P < 0.01, ^***^P < 0.001 vs. control.

To evaluate the correlation between HDGF and gefitinib efficacy in vivo, we conducted experiments in mice implanted with NSCLC cells with HDGF knockdown or overexpression. As shown in Fig. 4A–C, 50 mg/kg gefitinib treatment did not result in an obvious antitumor effect in H1975 xenografts. However, it caused significant tumor shrinkage in the HDGF sgRNA2 group, suggesting that HDGF knockdown improved gefitinib sensitivity. Conversely, in gefitinib-sensitive PC-9 xenografts, stable HDGF expression resulted in rapid tumor growth compared with the control group, but the tumor-promoting effect of HDGF was lessened by 10 mg/kg gefitinib administration (Fig. 4D–F). However, tumor regression occurred in both the HDGF overexpression and control groups, which may be due to the relatively high dosage of gefitinib used in this experiment because PC-9 cells are very sensitive to gefitinib. Nevertheless, HDGF overexpression still resulted in faster tumor growth than observed in the control group after gefitinib treatment (*P* < 0.05), implying that HDGF augmentation abrogated gefitinib efficacy to some extent. As shown in Fig. 4G, Ki-67 and HDGF expression in mouse tumor tissues exhibited similar patterns as tumor volume and weight in H1975 and PC-9 xenografts.

Because HDGF is a secretory protein, we detected its concentrations in the plasma of NSCLC tumor-bearing mice to explore whether plasma HDGF levels are relevant to gefitinib efficacy. As shown in Fig. 4H, I, mouse plasma HDGF concentration paralleled gefitinib efficacy. We also preliminarily analyzed plasma HDGF concentrations in eight NSCLC patients before and after taking gefitinib as a monotherapy. As shown in Fig. 4J, K, compared to the baseline, each of the cases and their average plasma HDGF levels became obviously elevated when drug resistance occurred. Furthermore, we measured plasma HDGF concentrations in five NSCLC patients before and after receiving AZD9291, a third-generation TKI, until they developed drug resistance. The results were similar to those observed in NSCLC patients with acquired gefitinib resistance (Fig. 4L, M). HDGF expression was detected in paired biopsy samples before and after TKI treatment. What’s exciting is that HDGF was highly expressed in both the cases when TKI resistance occurred, suggesting that higher HDGF levels may predict poor efficacy for TKI treatment. Nevertheless, only limited samples were tested in the present study, and more clinical samples are needed to verify this result.

### Mechanisms of HDGF-driven gefitinib resistance

**Fig. 5:**
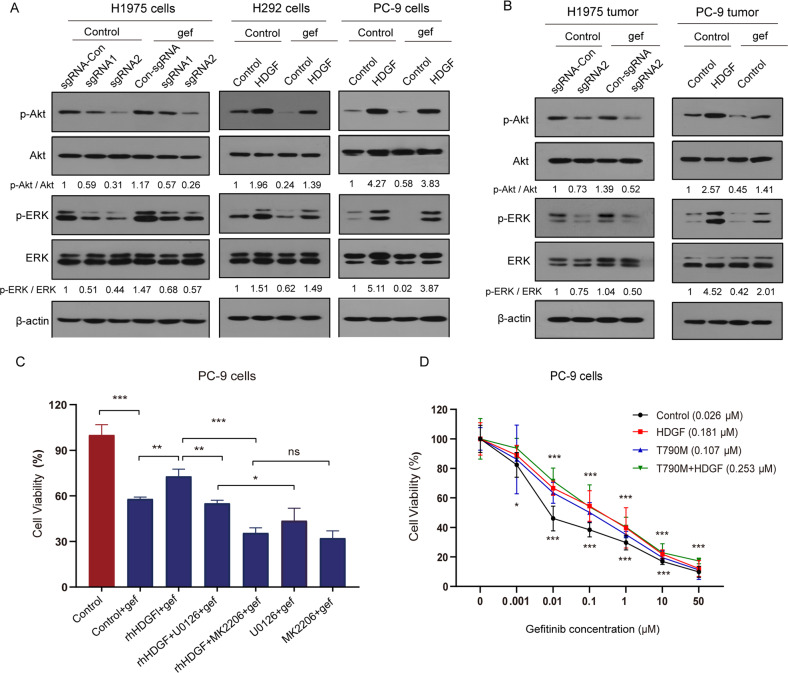
Mechanisms of HDGF-driven gefitinib resistance. The expression of p-Akt and p-ERK in NSCLC cells (**A**) or xenograft tumor tissues (**B**) with HDGF silencing or overexpression after gefitinib treatment. **C** Cell viability of PC-9 cells treated with 1 µM gefitinib for 24 h in the presence of rhHDGF, MK2206 (Akt inhibitor, 0.5 µM) or U0126 (ERK1/2 inhibitor, 5 µM). **D** The pcDNA3.1-EGFR T790M mutation plasmid and HDGF overexpression vector alone or together were introduced into the PC-9 cell, and then treated with gefitinib for 72 h. Data are from one representative experiment performed in triplicate. ^*^P < 0.05, ^**^P < 0.01, ^***^P < 0.001 vs. control.

To explore the mechanisms of HDGF-driven gefitinib resistance, we determined the gefitinib resistance-associated proteins p-Akt and p-ERK in NSCLC cells (Fig. 5A) and mouse tumor tissues (Fig. 5B). HDGF knockdown notably reduced p-Akt and p-ERK expression, and this effect was further strengthened by gefitinib, which alone increased Akt and ERK phosphorylation in H975 cells. In contrast, although gefitinib prominently inhibited p-Akt and p-ERK in H292 and PC-9 cells, it could not reverse the escalated Akt and ERK phosphorylation caused by HDGF overexpression. The above data further supported that HDGF levels were associated with gefitinib resistance-related signaling.

Previous results showed that HDGF-induced cell growth was abrogated or reversed by inhibitors of the Akt or ERK pathway. In Fig. 5C, we have added MK2206 (an inhibitor of Akt) or U0126 (an inhibitor of ERK) to validate whether HDGF promotes gefitinib-resistance via these two signal pathways. The results showed that HDGF treatment indeed decreased the sensitivity of PC-9 cells to gefitinib; however, the enhancing effects of HDGF on the gefitinib resistance were largely attenuated by MK2206 or U0126, suggesting that HDGF may induce gefitinib-resistance through Akt and ERK signal pathway.

EGFR T790M mutation is the primary mechanism of gefitinib resistance. To explore whether HDGF is involved in T790M mediated gefitinib resistance, the pcDNA3.1-EGFR T790M mutation plasmid and HDGF overexpression vector alone or together were introduced into the PC-9 cells. In Fig. 5D, our results showed that T790M mutation or HDGF overexpression alone resulted in gefitinib resistance with IC50 values of 0.107 µM and 0.181 µM, respectively. More importantly, when PC-9 cells were subjected to both T790M mutation and HDGF overexpression, gefitinib resistance (IC50 = 0.253 µM) was enhanced further than in the cells with single T790M mutation or HDGF overexpression. These results suggest that the gefitinib resistance caused by T790M mutation or HDGF overexpression may not share a same mechanism. Therefore, HDGF may be not involved in the gefitinib resistance initiated by T790M mutation.

### Crosstalk occurs between HDGF and EGFR

**Fig. 6:**
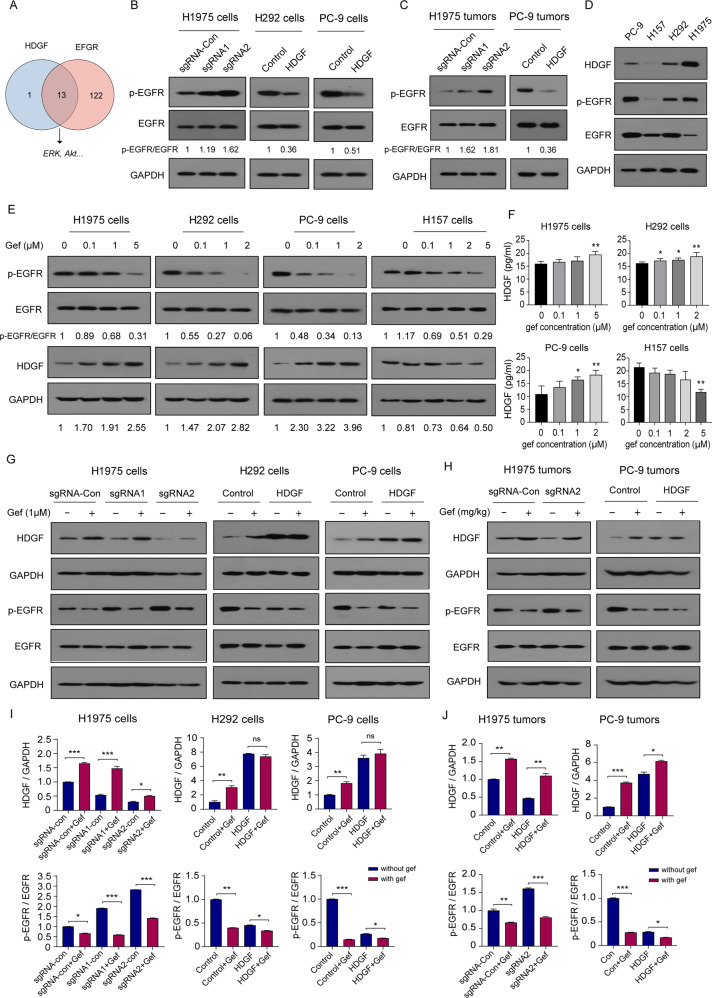
The complementary roles of HDGF and EGFR in NSCLC. **A** The overlapping pathways between HDGF and EGFR were retrieved from the GeneCards database. EGFR expression was detected in NSCLC cells (**B**) and xenograft tumors (**C**) with HDGF knockdown or overexpression. The expression levels of p-EGFR and HDGF were determined in NSCLC cell lines (**D**) and gefitinib-treated NSCLC cells for 24 h (**E**). **F** HDGF concentrations in the supernatants of NSCLC cells after gefitinib treatment for 24 h. Changes in HDGF and p-EGFR expression in gefitinib-treated NSCLC cells (**G**) and xenograft tumor tissues (**H**) with HDGF knockdown and overexpression. **I**, **J** Show the analytical results of **G**, **H**. Data are from one representative experiment performed in triplicate. ^*^P < 0.05, ^**^P < 0.01, ^***^P < 0.001 vs. control.

We found that silencing or overexpressing HDGF affected EGFR downstream Akt and ERK activation, which is related to TKI resistance. Then, we asked whether crosstalk exists between HDGF and EGFR. First, we searched Pathcards in the GeneCards database (http://www.genecards.org) and unsurprisingly found that the majority of HDGF pathways overlapped with EGFR, including Akt and ERK signaling (Fig. 6A). Next, we found that the levels of p-EGFR were inversely correlated with HDGF knockdown or overexpression in NSCLC cells and tumor tissues (Fig. 6B, C). Gefitinib inhibited p-EGFR but enhanced HDGF dose-dependently in tested NSCLC cells except H157 cells, which have extremely low p-EGFR and HDGF expression levels (Fig. 6D, E), indicating that gefitinib-induced HDGF elevation only occurred in EGFR-dependent NSCLC cells. In parallel, the concentrations of secreted HDGF in the supernatants of tested NSCLC cells except H157 cells were also increased after gefitinib treatment (Fig. 6F).

**Fig. 7:**
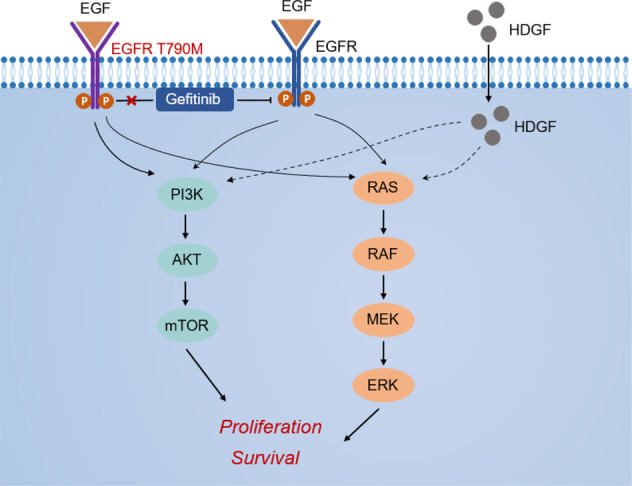
Schematic diagram of the contribution of HDGF to gefitinib resistance by activating EGFR downstream molecules as a bypass in NSCLC.

We then asked whether HDGF and EGFR have complementary roles in responding to gefitinib in NSCLC cells with HDGF loss and gain. As shown in Fig. 6G, I, HDGF knockdown caused a decrease in HDGF expression, and p-EGFR upregulation was reversed by gefitinib treatment in H1975 cells. In H292 and PC-9 cells, HDGF overexpression resulted in HDGF elevation and p-EGFR suppression, and gefitinib treatment further inhibited p-EGFR but without an obvious influence on HDGF expression. As shown in Fig. 6H, J, HDGF was increased by gefitinib administration in PC-9 xenograft tumors with HDGF overexpression (*P* < 0.05), and other changes in HDGF and p-EGFR in tumor tissues were consistent with the cell experiments. Therefore, the above data indicate crosstalk between HDGF and EGFR in NSCLC, and HDGF may serve as a bypass signaling molecule of EGFR to activate the downstream molecules Akt and ERK. Furthermore, HDGF and EGFR have complementary roles in maintaining the survival of tumor cells exposed to gefitinib. The function of HDGF in gefitinib resistance is illustrated by a schematic diagram in Fig. 7.

## Discussion

In the present study, we found that higher HDGF levels are not only associated with the malignant phenotype of NSCLC but can also induce gefitinib resistance. TKI resistance-related signaling, such as the PI3K/Akt and MEK/ERK pathways, could be bypass-activated by HDGF in an EGFR-independent manner. Furthermore, suppression of HDGF increased gefitinib efficacy in resistant NSCLC cells both in vitro and in vivo, suggesting that HDGF is a potential target to overcome gefitinib resistance.

First, our study demonstrated that HDGF promoted NSCLC tumorigenesis and facilitated metastasis. In H292 and PC-9 cells, which have relatively low levels of HDGF expression, forced HDGF expression increased the cells’ proliferation, colony formation, and migration and invasion abilities. Meanwhile, exogenous rhHDGF expression enhanced H292 and PC-9 cell proliferation, confirming the role of HDGF as a growth stimulator. The tumorigenesis function of HDGF was also confirmed in HDGF-overexpressing PC-9 xenograft mice. In contrast, silencing HDGF by CRISPR inhibited the malignant phenotype of H1975 cells and retarded tumor development in vivo. Studies have shown that targeting HDGF by miRNAs or antibodies is effective in inhibiting the growth of NSCLC [16, 19–21] or lung squamous cell carcinoma [30]. However, until now, most studies on HDGF have mainly focused on its tumorigenic role, while only limited attention has been given to determining its function in anticancer therapy resistance. As reported, HDGF participated in the development of chemotherapy drug resistance in colorectal cancer [25] and tongue squamous cell carcinoma [26] or promoted radioresistance in breast cancer [27]. Inhibiting HDGF with the green tea polyphenol (-)-epigallocatechin-3-gallate (EGCG) could increase the NSCLC response to chemotherapy [31].

Gefitinib is an extensively used first-generation EGFR-TKI for NSCLC patients with EGFR-sensitive mutations, but inevitable drug resistance hampers its clinical benefits. The major mechanisms accounting for TKI resistance include alterations in drug targets, such as T790M, which accounts for half of all cases of acquired resistance, while bypass signaling activation comprises 20 to 25% of all cases [32]. However, there are still approximately 15–20% of NSCLC patients in whom no mechanisms for acquired TKI resistance have been identified [29]. Our results revealed that HDGF knockdown could increase gefitinib sensitivity, while HDGF augmentation abrogated gefitinib efficacy to some extent in NSCLC. In detail, knockdown of HDGF inhibited tumor growth in H1975 cells in vitro and in vivo, while overexpression of HDGF retarded the gefitinib response in PC-9 cells. Immunohistochemical staining of Ki-67 and HDGF in mouse tumor tissue sections was consistent with the above results. Therefore, our data indicated that HDGF expression was correlated with gefitinib efficacy in NSCLC. These results were partly supported by a recent study that provides some clues for HDGF-S165 and TKI resistance via site-specific peptide reporters and targeted mass spectrometry at the nanogram level in cells [28].

High serum HDGF levels predicted bone metastasis and unfavorable prognosis in NSCLC [13]. First, we found a parallel correlation between plasma HDGF levels and the antitumor effect of gefitinib in NSCLC xenografts. In a small number of clinical samples, the HDGF levels were also elevated in the peripheral blood of NSCLC patients who took gefitinib and AZD9291 after drug resistance occurred. Meanwhile, HDFG overexpression was associated with gefitinib resistance in clinical samples. Thus, HDGF is involved in gefitinib resistance, and HDGF levels in plasma or tissue samples may predict TKI efficacy to some extent. However, only limited clinical samples were tested in the present study; thus, more clinical specimens are needed to verify these results.

PI3K/Akt and MAPK/ERK signaling are major EGFR downstream pathways leading to cell proliferation. TKIs bind to the tyrosine kinase domain of EGFR and block EGFR-driven signaling downstream to exert their tumor-inhibitory effects. The EGFR T790M mutation interferes with the interaction by enhancing its affinity to ATP to confer gefitinib resistance [33]. In EGFR-independent conditions, dysregulation of other receptor tyrosine kinases or abnormal activation of downstream signals has a compensatory function to abrogate EGFR inhibition and results in TKI resistance [3]. The bypass signaling pathway shares the same downstream pathways with EGFR and converges to trigger the PI3K/Akt and MAPK/ERK signaling axes [30, 34]. A great number of studies have demonstrated aberrant activation of p-Akt and p-ERK when EGFR-TKI resistance occurs [29], but restraining p-Akt and p-ERK expression could recover gefitinib sensitivity in NSCLC [35, 36]. In the present study, HDGF knockdown inhibited Akt and ERK phosphorylation, while HDGF overexpression exerted the opposite effect, suggesting that it regulates p-Akt and p-ERK activation. In gefitinib-sensitive NSCLC cells, gefitinib-restrained p-Akt and p-ERK levels were abrogated by HDGF overexpression; however, in resistant H1975 cells, the activation of Akt and ERK was strongly suppressed by HDGF depletion. Inhibitors of Akt or ERK were used to validate whether HDGF inducing cell growth and gefitinib resistance by these two pathways. These data indicated that HDGF could enhance EGFR downstream p-Akt and p-ERK signaling, which is related to gefitinib resistance.

As both EGFR and HDGF regulate the activation of Akt and ERK, we were not surprised to find that the majority of known pathways of HDGF overlapped with EGFR, including Akt and ERK signaling, by searching GeneCards (https://www.genecards.org). This is consistent with a study in ovarian cancer cells in which extracellular HDGF stimulated the phosphorylation of ERK [37] and promoted tumor development and progression by regulating the PI3K-Akt pathway in bladder cancer cells [38]. Tumor cells usually orchestrate a network of signaling pathways to sustain their survival when facing death caused by anticancer therapy. When the dominant pathway, such as EGFR, is inhibited by a TKI, tumor cells tend to turn on the critical downstream effector through bypass signaling pathways, maintaining continued cell survival and growth [39]. In the present study, we found that HDGF and p-EGFR expression levels were complementary to each other in NSCLC cells with or without HDGF loss and gain. Gefitinib treatment could reduce EGFR phosphorylation but increase HDGF expression, aberrantly activating the PI3K/Akt and MEK/ERK pathways. However, knockdown of HDGF in H1975 cells could reactivate EGFR and decrease HDGF when exposed to gefitinib, suggesting both a delicate balance and complementarity among HDGF and p-EGFR in EGFR-dependent NSCLC cells when exposed to gefitinib. Considering all the relevant information, the increase of HDGF levels may serve as a bypass survival signal to trigger resistance to gefitinib, and drug resistance can be overcome by silencing HDGF.

In summary, the present research found that HDGF not only promoted the NSCLC cell malignant phenotype but also contributed to gefitinib resistance. As a bypass and compensatory signaling pathway, HDGF activates the EGFR downstream PI3K/Akt and MEK/ERK pathways, which are associated with gefitinib resistance. Targeting HDGF could restore gefitinib sensitivity through a delicate balance between HDGF expression and EGFR reactivation as well as their shared downstream molecules. Therefore, HDGF can be regarded as a potential underlying mechanism of gefitinib resistance and is a promising target both against NSCLC and to increase TKI efficacy in NSCLC.

## Materials and methods

### Cell lines and culture

Human NSCLC cell lines H1975, H157, H460, H292 and PC-9 were purchased from American Type Culture Collection (Manassas, Virginia, USA), while A549 and H1650 cells were obtained from National Infrastructure of Cell Line Resource (Beijing, China). All cells were maintained in RPMI-1640 with 10% fetal bovine serum (FBS) at 37 °C in a humidified atmosphere of 5% CO_2_.

### Drugs and reagents

Gefitinib was obtained from AstraZeneca (Cheshire, UK). Antibodies against Akt (9272), ERK1/2 (9102) and p-ERK1/2 (T202/Y204) (9101) were purchased from Cell Signaling Technology (Beverly, MA). HDGF and p-Akt (Ser473) were obtained from Abcam (Cambridge, UK). EGFR and p-EGFR were purchased from ABclonal (Wuhan, China). GAPDH, β-actin, β-tubulin and Ki-67 were purchased from TDYbio (Beijing, China). Recombinant human HDGF (rhHDGF) was obtained from ProSpec-Tany TechnoGene Ltd. (Israel).

### Establishment of stably transfected NSCLC cells

The recombinant HDGF plasmid in the pLenti6/TR lentiviral vector (Invitrogen) and the pcDNA3.1-EGFR T790M mutation plasmid were obtained by molecular cloning. The single-guide RNA (sgRNA) for HDGF was ligated into the lentiCRISPRv2 plasmid. NSCLC cells were transfected with plasmid and selected to generate a stable cell line for further study. The sequences for HDGF sgRNA1, sgRNA2, control sgRNA (sgRNA-Con) and other primers are listed in Tables S1 and S2.

### Quantitative RT‒PCR (qRT‒PCR)

Total RNA was extracted using TRIzol reagent (Invitrogen, Carlsbad, CA, USA). qRT‒PCR was performed as previously described [40]. The primer sequences of HDGF and GAPDH are presented in Table S2.

### Cell proliferation assay

The influence of HDGF knockdown or overexpression, as well as the induction of rhHDGF on NSLCL cell growth, was determined by counting cell numbers. The MTT assay was used to assess cell growth inhibition following gefitinib treatment. Briefly, 1 × 10^4^ cells in 96-well plates were treated with gefitinib (0.001 ~ 50 μM) for 72 h. The optical density at 570 nm was measured, and the IC50 values were calculated by GraphPad Prism 6.0 software (San Diego, CA).

### Colony formation assay

Cells were seeded in 6 cm culture dishes at 200 cells per dish. After 14 days of incubation, cells were fixed in 4% paraformaldehyde and stained with crystal violet, and colonies (diameter > 40 μm) were counted and compared.

### Transwell assay

Experiments were performed in a 24-well Boyden chamber as previously described [40]. Cells were used at a density of 3 × 10^4^ for migration and invasion assays without or with Matrigel. Photographs of four randomly selected fields were taken, and cell numbers were counted under a microscope.

### Western blot

Immunoblotting was carried out as described previously [22]. Protein bands were visualized using an enhanced chemiluminescence kit, and their intensities were measured by ImageJ software.

### In vivo study of the correlation between HDGF and gefitinib efficacy

All animals were obtained from Beijing HFK Bioscience Co. Ltd., (China), and were randomly divided into experimental groups using the random number generated by Excel. NSCLC xenografts were established by subcutaneously inoculating cells into 6–8 weeks male BALB/c nude mic (SCXK-2016-006). When the tumor volumes reached approximately 50–100 mm^3^, the mice were randomly divided and treated as described below. At the end of the test, the mice were sacrificed, and plasma and tumors were collected for further analysis.

Mice were divided into three groups and implanted with H1975 cells (5 × 10^5^) with sgRNA control, sgRNA1 or sgRNA2. Other mice were inoculated with PC-9 cells (8 × 10^5^) overexpressing HDGF or its vector control. The effect of HDGF knockdown on gefitinib efficacy in H1975 cells was evaluated in four groups of mice: sgRNA-control or sgRNA2 treated with gefitinib (50 mg/kg) or vehicle. The effect of HDGF overexpression on gefitinib efficacy in PC-9 cells was assessed in four groups: vector control or HDGF overexpression treated with gefitinib (10 mg/kg) or vehicle. The data of tumor volume was collected in a blind manner. The student who measured the tumor volume had no idea of the group information.

### ELISA

HDGF concentrations in mouse plasma were evaluated by a kit from Enzyme-linked Biotechnology Co., Ltd. (Shanghai, China). HDGF levels in the plasma of NSCLC patients were determined by an ELISA kit from Anrc (Tianjin, China).

### Immunohistochemistry

The immunohistochemistry (IHC) procedure was performed as described elsewhere. The tissue sections were incubated with antibodies against HDGF or Ki-67. A peroxidase-conjugated goat anti-rabbit secondary antibody from Jackson (West Grove, PA) was diluted in PBS. Specific binding was visualized with the peroxidase substrate diaminobenzidine (DAB) from Pierce (Rockford, IL).

### Statistical analysis

The data are presented as the means ± SDs. For the in vitro studies, experiments were performed at least in triplicates. For the in vivo studies, 5 mice were included in each group. The data which were out of the ranges of the means ± 3 × SDs were excluded following the pre-established criteria. The IC50 values were determined by fitting the data in GraphPad Prism 6.0. The Mann‒Whitney U test was performed to compare gene expression between different groups, and other comparisons were made using Student’s t-test. P-values < 0.05 were considered statistically significant. Data variances were compared using F tests. Mann Whitney test was used to compare the data when *p*-value of F tests is smaller than 0.05. All statistical analyses were performed with SPSS 18.0 (SPSS, Chicago, IL).

## Supplementary information

Combined supplementary materials in one flie

Original Western Blots

## Data Availability

The datasets used and/or analyzed during the current study are available from the corresponding author upon reasonable request.
