# Supplementary material for: HDGF promotes gefitinib resistance by activating the PI3K/AKT and MEK/ERK signaling pathways in non-small cell lung cancer
Source: Cell Death Discov. 2023 Jun 10;9:181. doi: 10.1038/s41420-023-01476-0 (PMC10257651; doi:10.1038/s41420-023-01476-0)
Supplement: Supplementary file 1 — Combined supplementary materials in one flie [file 41420_2023_1476_MOESM1_ESM.pdf]

Table S1. The sequence of single-guide RNA (sgRNA) for HDGF

SgRNA name	Sequence
HDGF-sgRNA1	5’-GGAGTACAAATGCGGGGACC-3’
HDGF- sgRNA2	5’-ACGTCCACACTTAACTGCGC-3’
sgRNA-Con	5’-TTCTCCGAACGTGTCACGTT-3’

sgRNA-Con, non-targeting.

Table S2. The sequence of primers used in the present study

Primer name	Sequence
HDGF-ORF-PF2-BamHI	CGGGATCCATGCACCCGGAAGGTGGCCAATT
HDGF-ORF-PF3-BamHI	CGGGATCCATGGAGCAGAGGGCAGGCGGAAA
HDGF-ORF-PR/XholI	CCGCTCGAGCTACAGGCTCTCATGATCTCTGA
CRISPR-HDGF-1-sense	CACCGGGAGTACAAATGCGGGGACC
CRISPR-HDGF-1-anti-sense	AAACGGTCCCCGCATTTGTACTCCC
CRISPR-HDGF-2-sense	CACCGACGTCCACACTTAACTGCGC
CRISPR-HDGF-2-anti-sense	AAACGCGCAGTTAAGTGTGGACGTC
HDGF sense	5’-AGAACAACCCTACTGTCAAG-3’
HDGF anti-sense	5’-CTCTTCAACGCTCCTTT-3’
GAPDH sense	5’-GACAGTCAGCCGCATCTTC-3’
GAPDH anti-sense	5’-CAACAATATCCACTTTACCAG-3’

Fig S1. HDGF was identified as a potential molecule accounting for gefitinib resistance. (**A**) Differentially expressed proteins in H1975 cells after *Marsdenia tenacissima* extract (MTE) combined with gefitinib treatment, which was detected by 2D-gel electrophoresis coupled with high-performance liquid chromatography-tandem mass spectrometry (HPLC–MS/MS). (**B**) Western blotting validated HDGF expression was decreased by MTE combined with gefitinib in H1975 cells. (**C**) shows the analytical results of B. (**D**) HDGF expression levels in various NSCLC cell lines. (**E**) shows the analytical results of D.

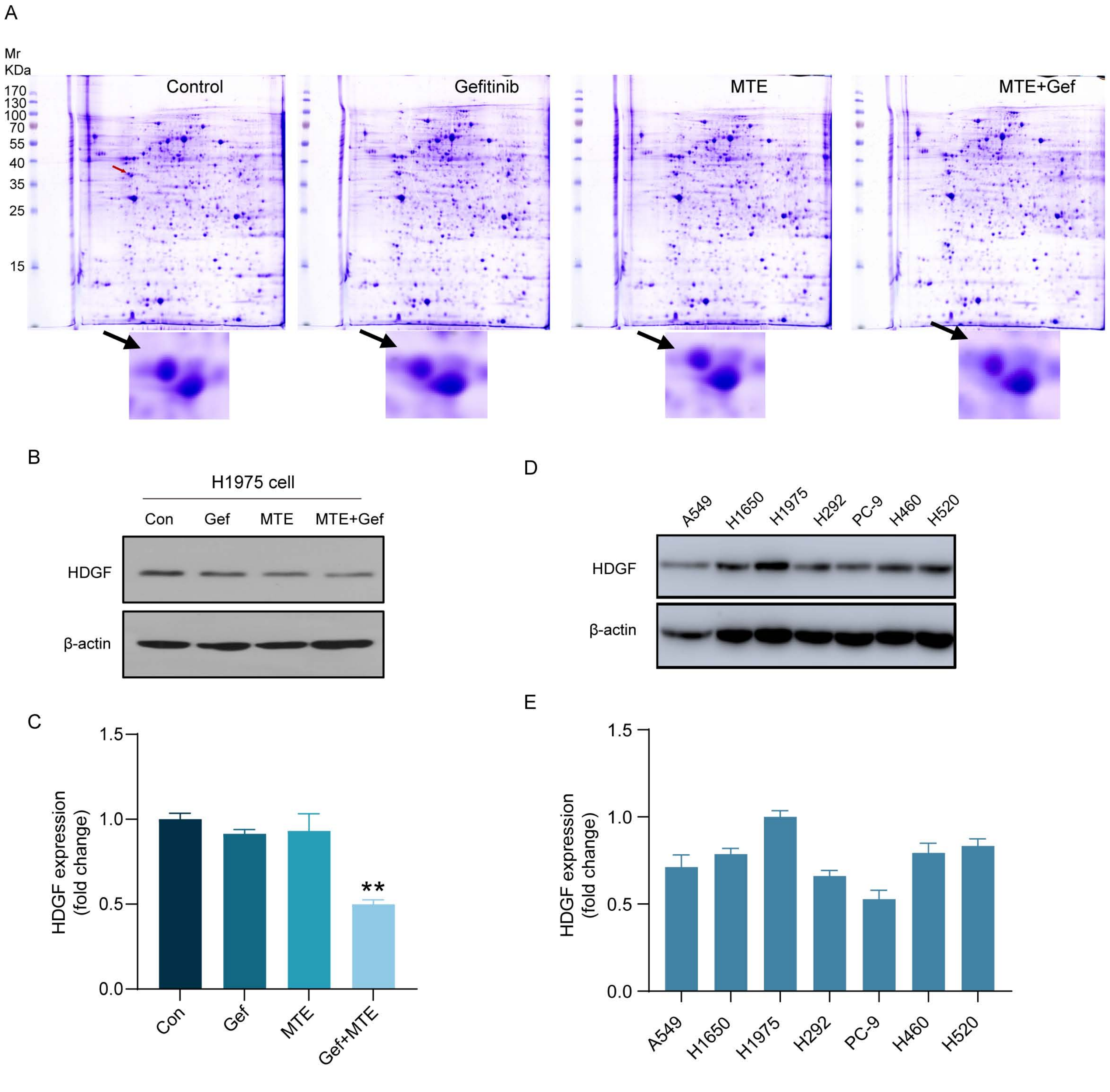

A
Mr
KDa
170
130
100
70
55
40
35
25
15
Control
Gefitinib
MTE
MTE+Gef
B
H1975 cell
Con
Gef
MTE
MTE+Gef
HDGF
β-actin
C
HDGF expression (fold change)
1.5
1.0
0.5
0.0
**
Con
Gef
MTE
Gef+MTE
D
A549
H1650
H1975
H292
PC-9
H460
H520
HDGF
β-actin
E
HDGF expression (fold change)
1.5
1.0
0.5
0.0
A549
H1650
H1975
H292
PC-9
H460
H520
