# Supplementary material for: HDGF promotes gefitinib resistance by activating the PI3K/AKT and MEK/ERK signaling pathways in non-small cell lung cancer
Source: Cell Death Discov. 2023 Jun 10;9:181. doi: 10.1038/s41420-023-01476-0 (PMC10257651; doi:10.1038/s41420-023-01476-0)
Supplement: Supplementary file 2 — Original Western Blots [file 41420_2023_1476_MOESM2_ESM.pptx]

## Slide 1
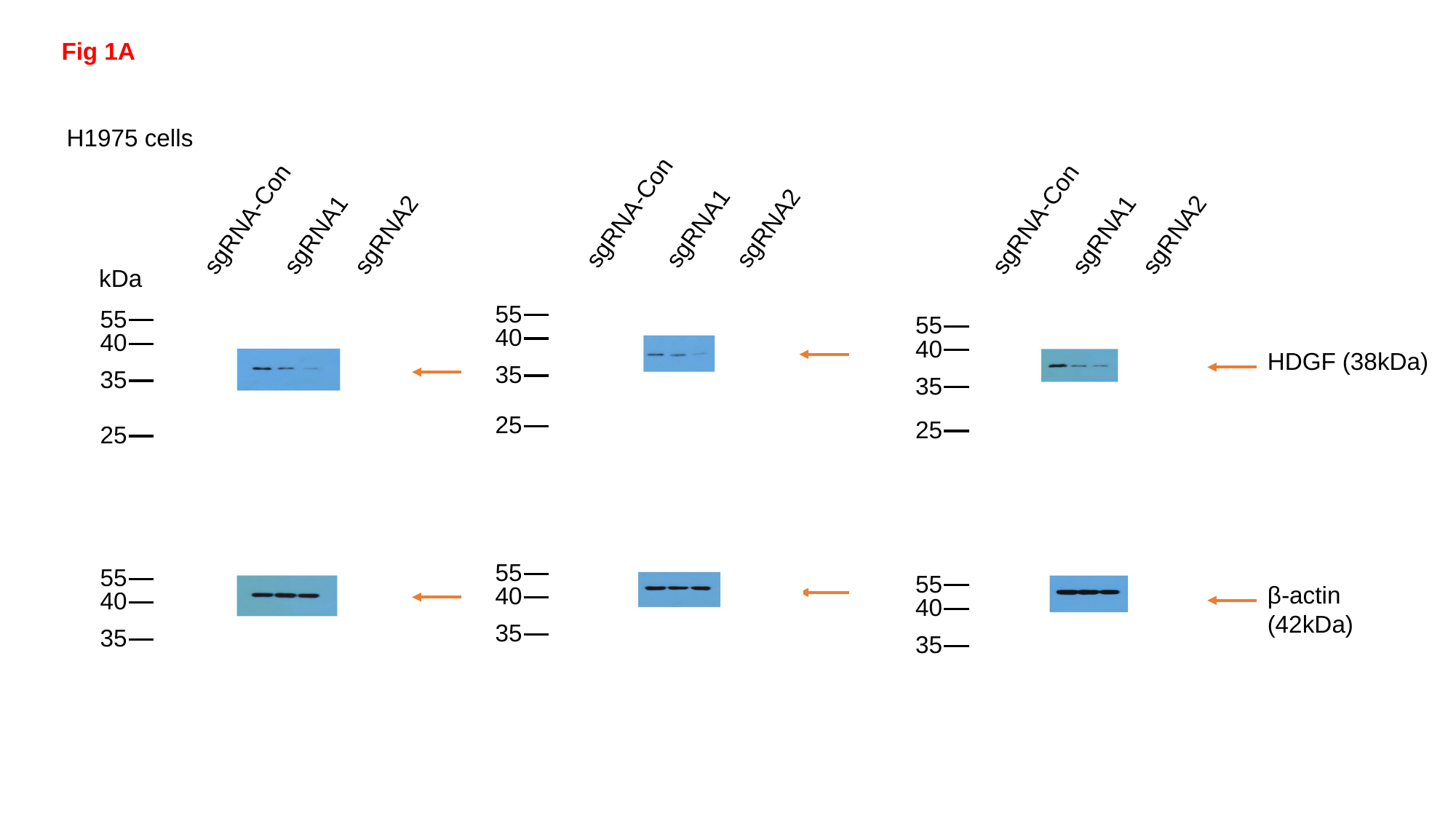

Fig 1A
H1975 cells
sgRNA-Con
sgRNA1
sgRNA2
sgRNA-Con
sgRNA1
sgRNA2
sgRNA-Con
sgRNA1
sgRNA2
kDa
 55
 55
 55
 40
 40
 40
HDGF (38kDa)
 35
 35
 35
 25
 25
 25
 55
 55
 55
β-actin (42kDa)
 40
 40
 40
 35
 35
 35

## Slide 2
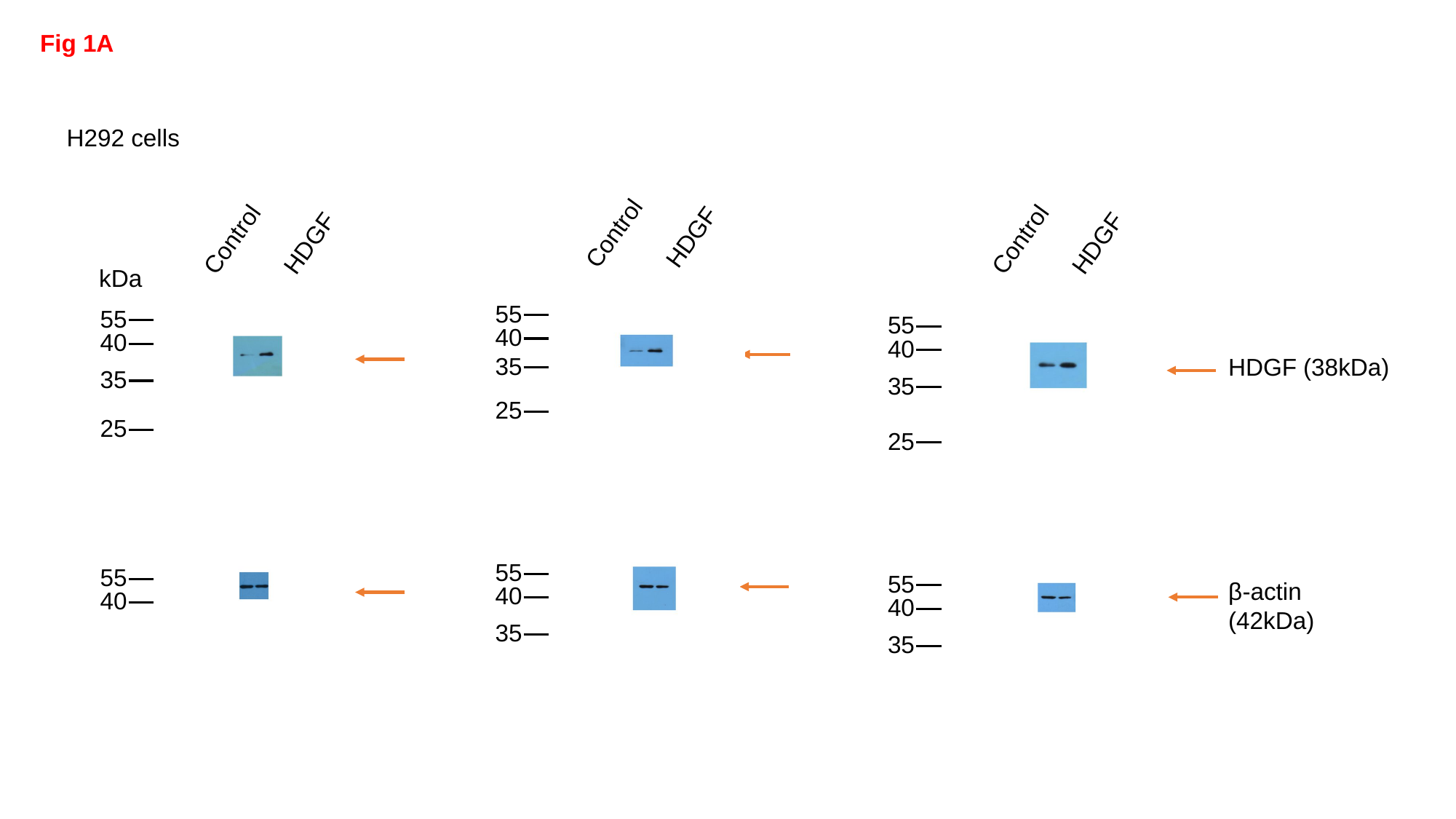

Fig 1A
H292 cells
Control
HDGF
Control
HDGF
Control
HDGF
kDa
 55
 55
 55
 40
 40
 40
 35
HDGF (38kDa)
 35
 35
 25
 25
 25
 55
 55
 55
β-actin (42kDa)
 40
 40
 40
 35
 35

## Slide 3
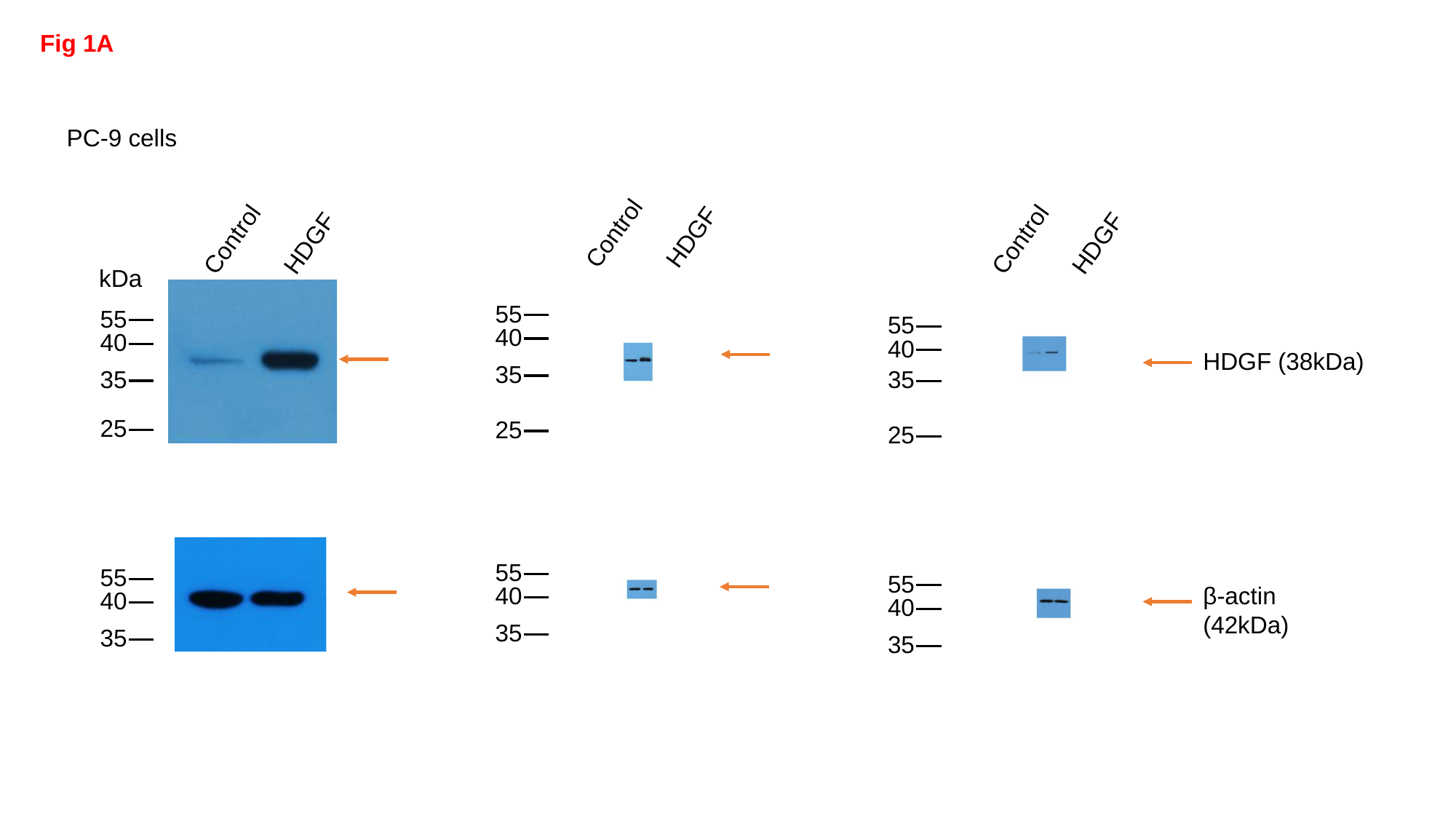

Fig 1A
PC-9 cells
Control
HDGF
Control
HDGF
Control
HDGF
kDa
 55
 55
 55
 40
 40
 40
HDGF (38kDa)
 35
 35
 35
 25
 25
 25
 55
 55
 55
 40
β-actin (42kDa)
 40
 40
 35
 35
 35

## Slide 4
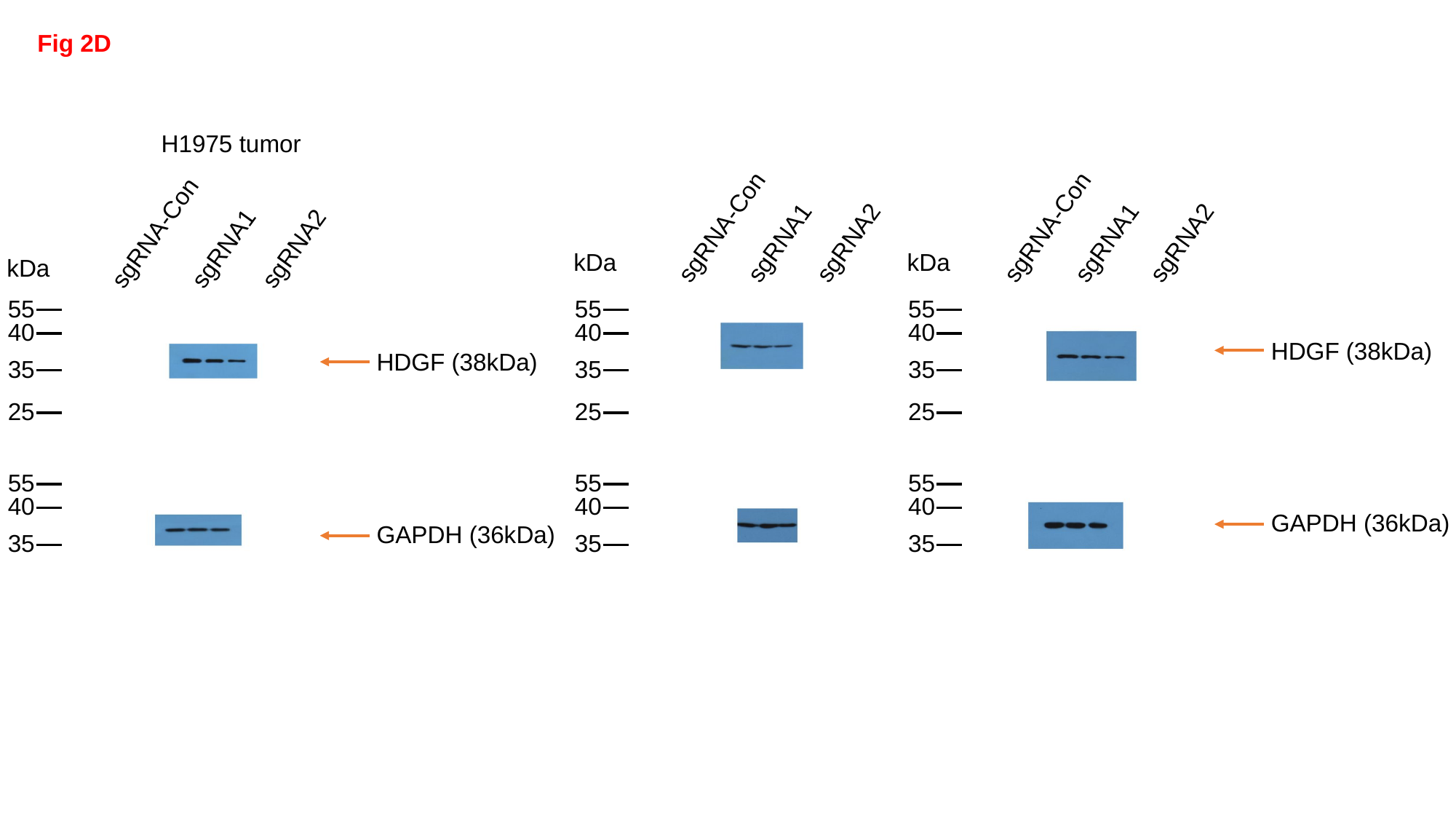

Fig 2D
H1975 tumor
sgRNA-Con
sgRNA1
sgRNA2
sgRNA-Con
sgRNA1
sgRNA2
sgRNA-Con
sgRNA1
sgRNA2
kDa
kDa
kDa
 55
 55
 55
 40
 40
 40
HDGF (38kDa)
HDGF (38kDa)
 35
 35
 35
 25
 25
 25
 55
 55
 55
 40
 40
 40
GAPDH (36kDa)
GAPDH (36kDa)
 35
 35
 35

## Slide 5
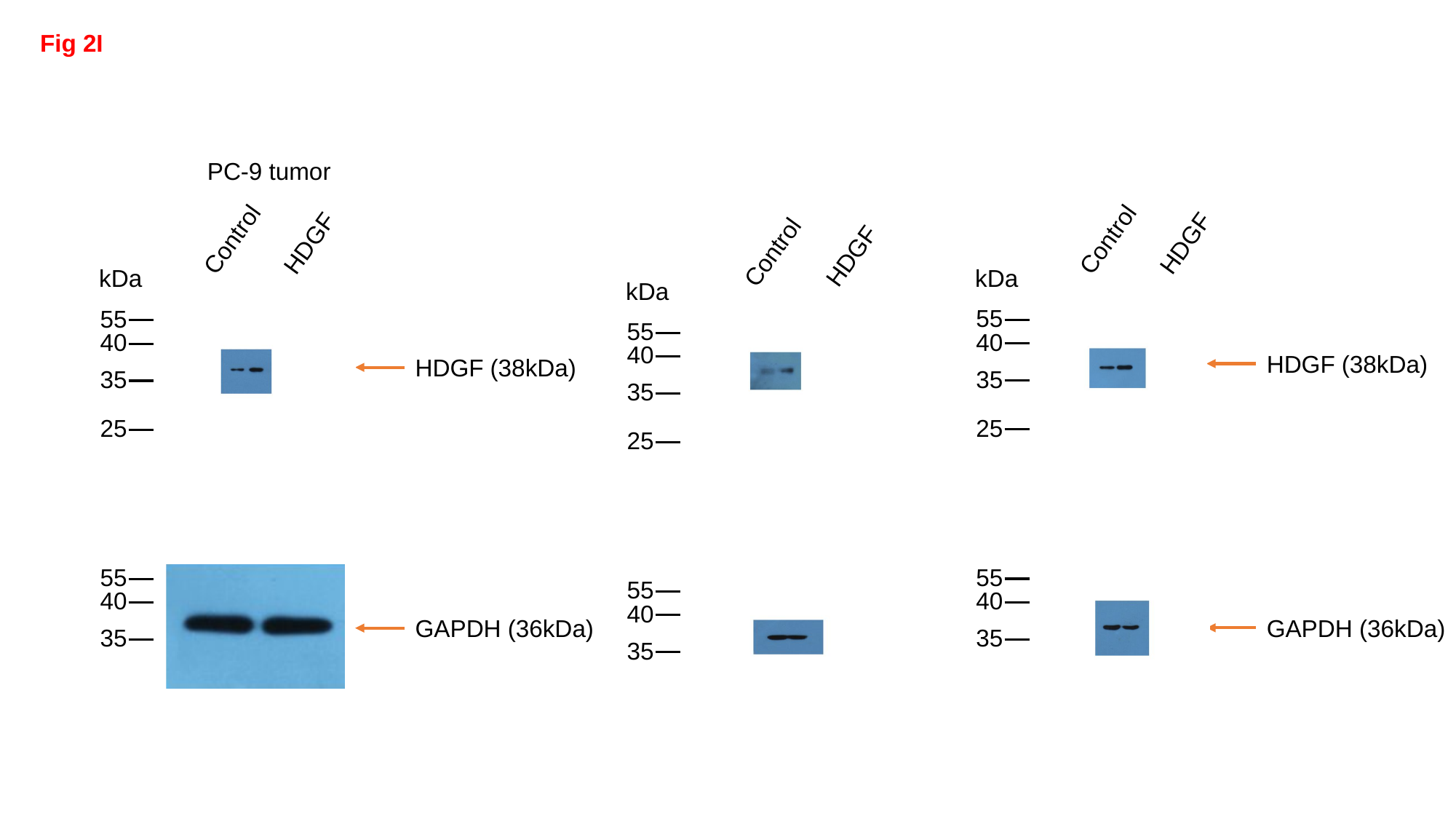

Fig 2I
PC-9 tumor
Control
HDGF
Control
HDGF
Control
HDGF
kDa
kDa
kDa
 55
 55
 55
 40
 40
 40
HDGF (38kDa)
HDGF (38kDa)
 35
 35
 35
 25
 25
 25
 55
 55
 55
 40
 40
 40
GAPDH (36kDa)
GAPDH (36kDa)
 35
 35
 35

## Slide 6
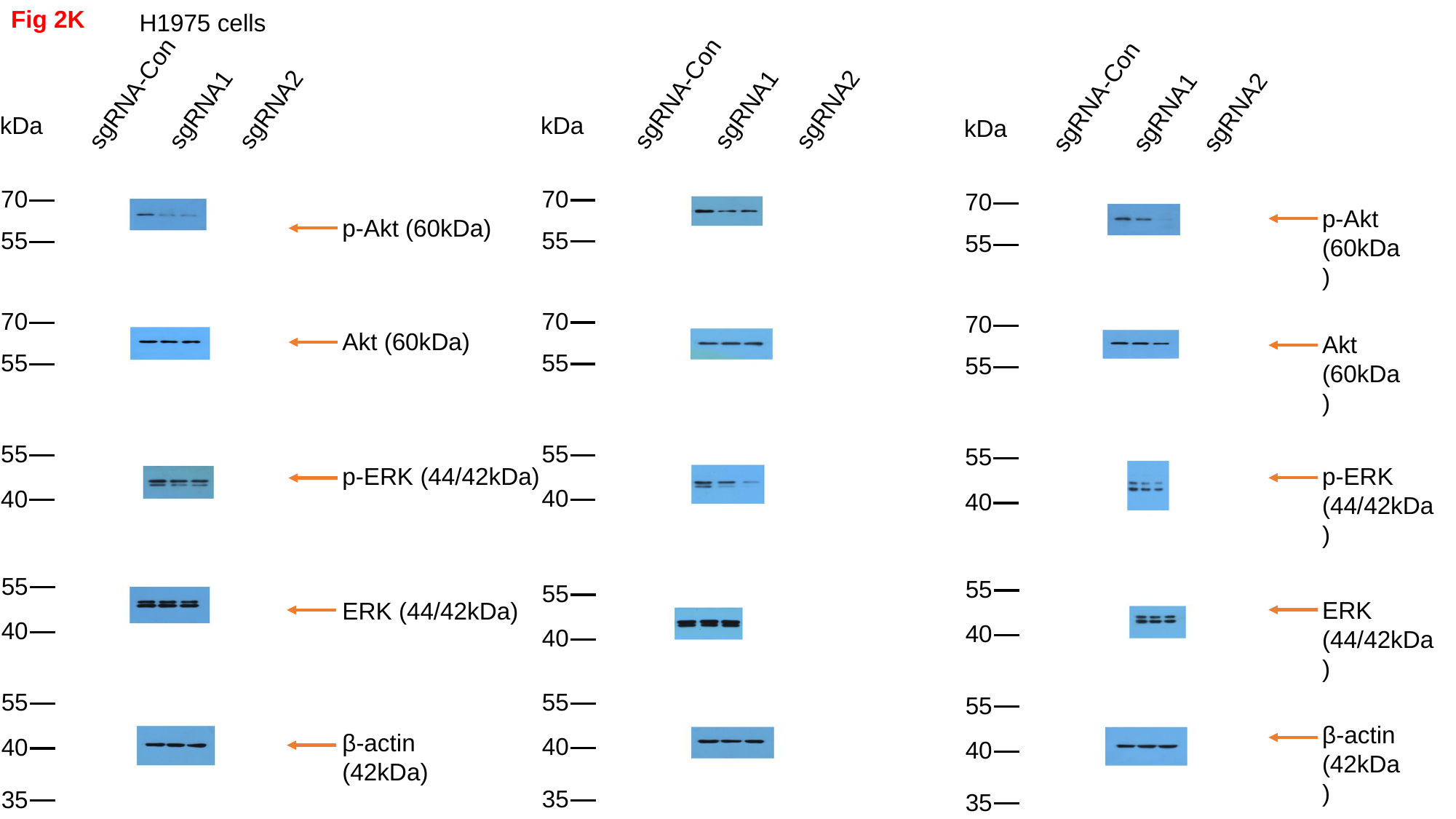

Fig 2K
H1975 cells
sgRNA-Con
sgRNA1
sgRNA2
sgRNA-Con
sgRNA1
sgRNA2
sgRNA-Con
sgRNA1
sgRNA2
kDa
kDa
kDa
 70
 70
 70
p-Akt
(60kDa)
p-Akt (60kDa)
 55
 55
 55
 70
 70
 70
Akt (60kDa)
Akt
(60kDa)
 55
 55
 55
 55
 55
 55
p-ERK
(44/42kDa)
p-ERK (44/42kDa)
 40
 40
 40
 55
 55
 55
ERK
(44/42kDa)
ERK (44/42kDa)
 40
 40
 40
 55
 55
 55
β-actin
(42kDa)
β-actin (42kDa)
 40
 40
 40
 35
 35
 35

## Slide 7
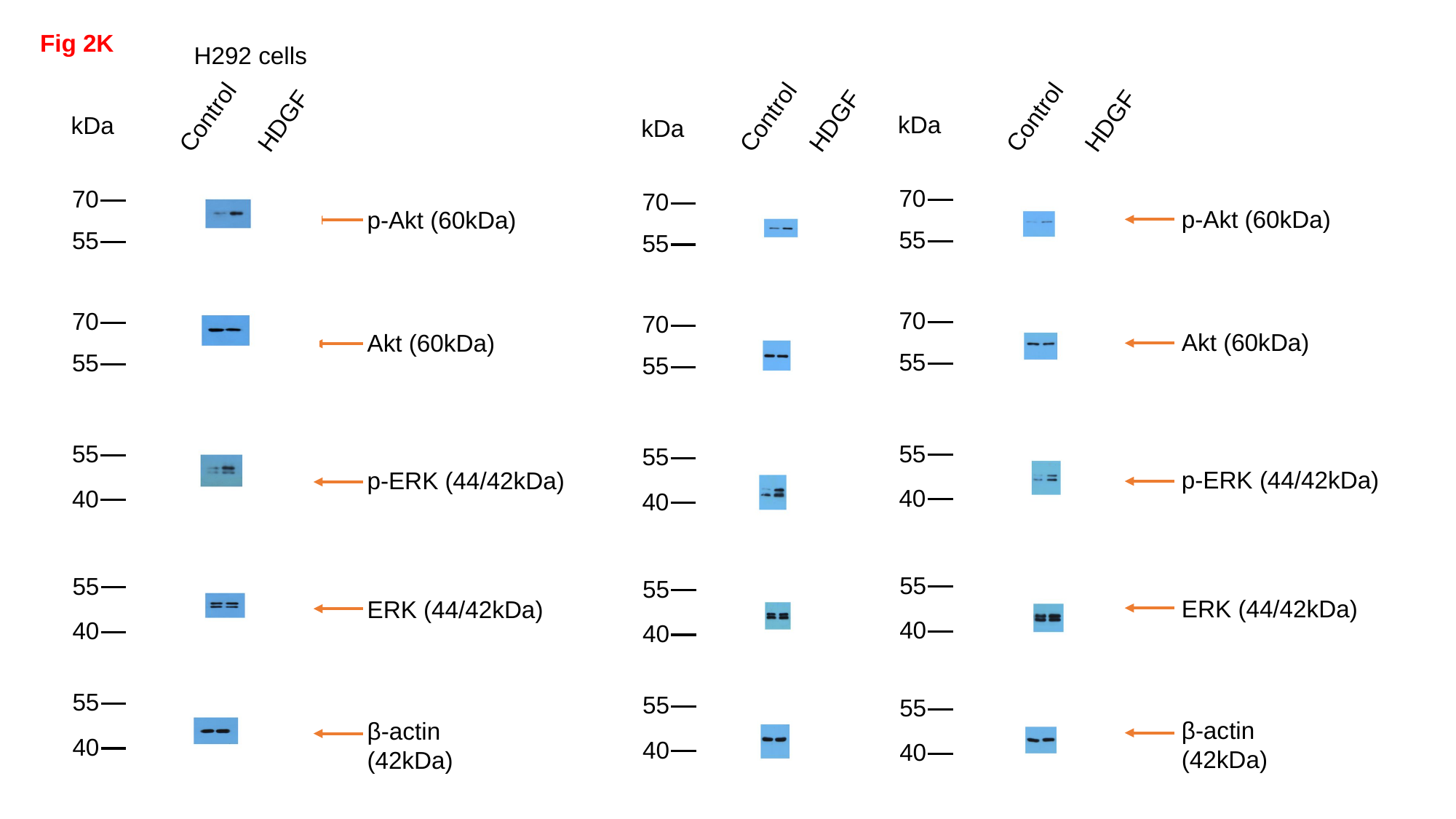

Fig 2K
H292 cells
Control
Control
Control
HDGF
HDGF
HDGF
kDa
kDa
kDa
 70
 70
 70
p-Akt (60kDa)
p-Akt (60kDa)
 55
 55
 55
 70
 70
 70
Akt (60kDa)
Akt (60kDa)
 55
 55
 55
 55
 55
 55
p-ERK (44/42kDa)
p-ERK (44/42kDa)
 40
 40
 40
 55
 55
 55
ERK (44/42kDa)
ERK (44/42kDa)
 40
 40
 40
 55
 55
 55
β-actin (42kDa)
β-actin (42kDa)
 40
 40
 40

## Slide 8
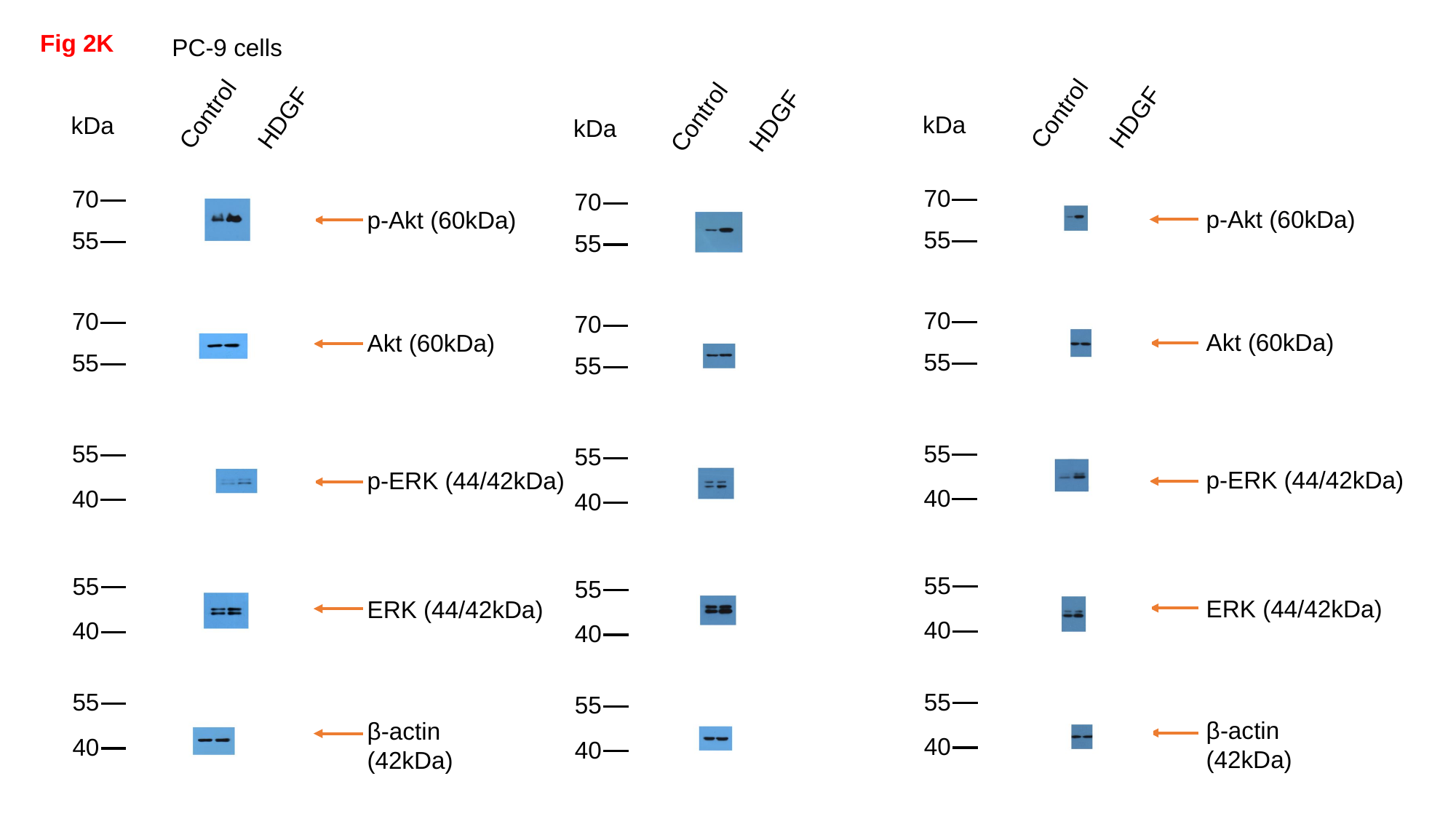

Fig 2K
PC-9 cells
Control
Control
Control
HDGF
HDGF
HDGF
kDa
kDa
kDa
 70
 70
 70
p-Akt (60kDa)
p-Akt (60kDa)
 55
 55
 55
 70
 70
 70
Akt (60kDa)
Akt (60kDa)
 55
 55
 55
 55
 55
 55
p-ERK (44/42kDa)
p-ERK (44/42kDa)
 40
 40
 40
 55
 55
 55
ERK (44/42kDa)
ERK (44/42kDa)
 40
 40
 40
 55
 55
 55
β-actin (42kDa)
β-actin (42kDa)
 40
 40
 40

## Slide 9
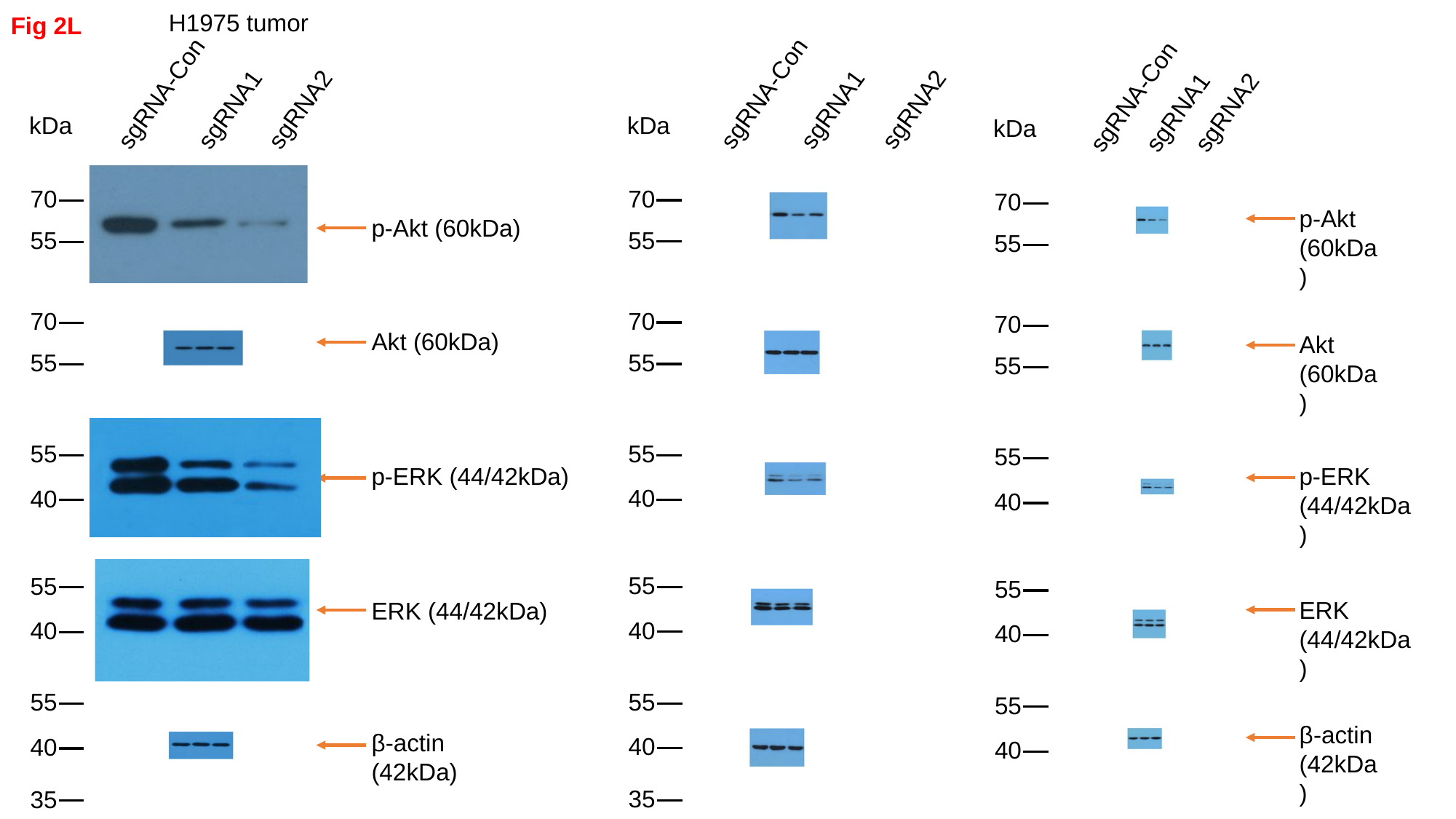

H1975 tumor
Fig 2L
sgRNA-Con
sgRNA1
sgRNA2
sgRNA-Con
sgRNA1
sgRNA2
sgRNA-Con
sgRNA1
sgRNA2
kDa
kDa
kDa
 70
 70
 70
p-Akt
(60kDa)
p-Akt (60kDa)
 55
 55
 55
 70
 70
 70
Akt (60kDa)
Akt
(60kDa)
 55
 55
 55
 55
 55
 55
p-ERK
(44/42kDa)
p-ERK (44/42kDa)
 40
 40
 40
 55
 55
 55
ERK
(44/42kDa)
ERK (44/42kDa)
 40
 40
 40
 55
 55
 55
β-actin
(42kDa)
β-actin (42kDa)
 40
 40
 40
 35
 35

## Slide 10
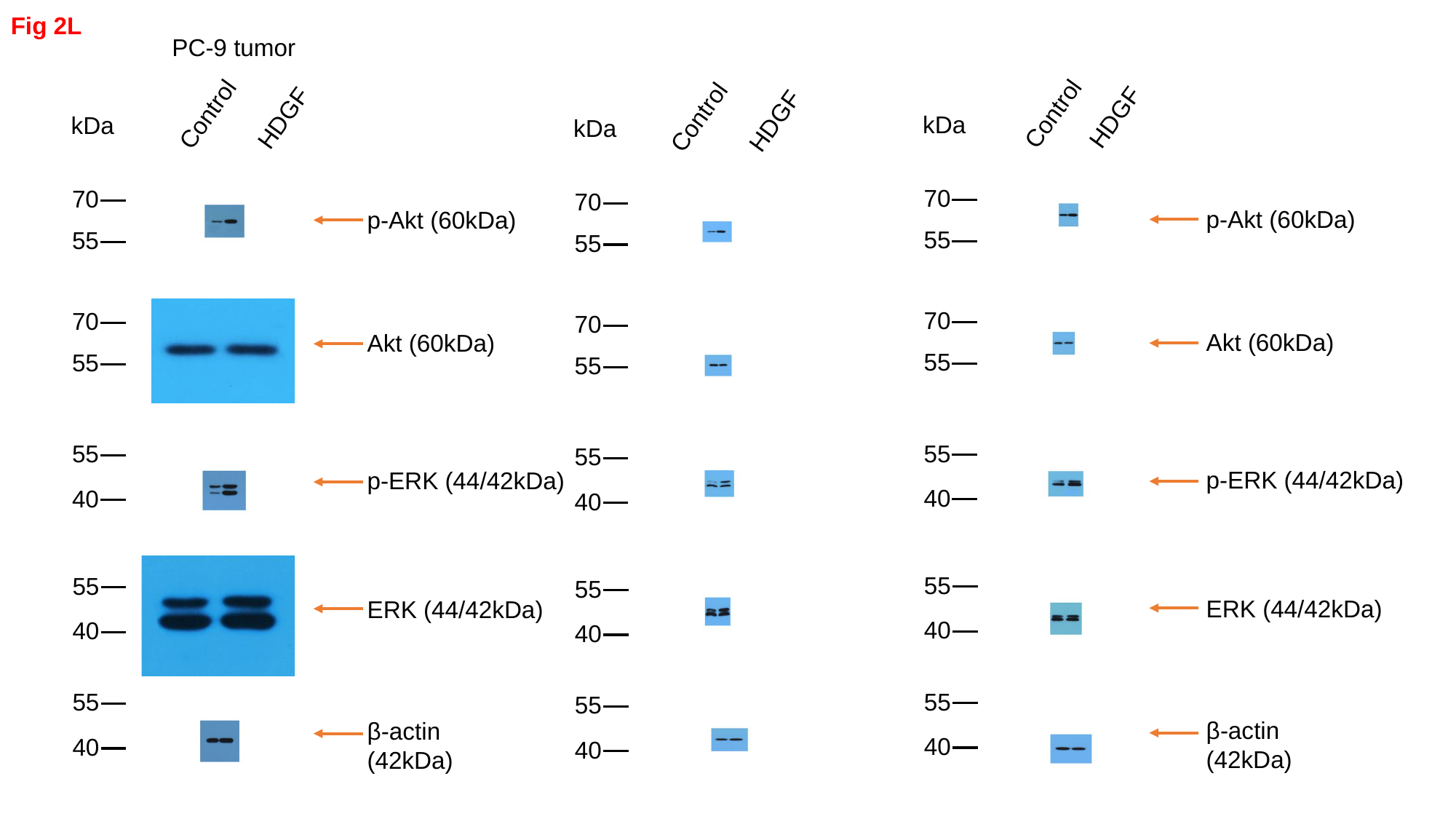

Fig 2L
PC-9 tumor
Control
Control
Control
HDGF
HDGF
HDGF
kDa
kDa
kDa
 70
 70
 70
p-Akt (60kDa)
p-Akt (60kDa)
 55
 55
 55
 70
 70
 70
Akt (60kDa)
Akt (60kDa)
 55
 55
 55
 55
 55
 55
p-ERK (44/42kDa)
p-ERK (44/42kDa)
 40
 40
 40
 55
 55
 55
ERK (44/42kDa)
ERK (44/42kDa)
 40
 40
 40
 55
 55
 55
β-actin (42kDa)
β-actin (42kDa)
 40
 40
 40

## Slide 11
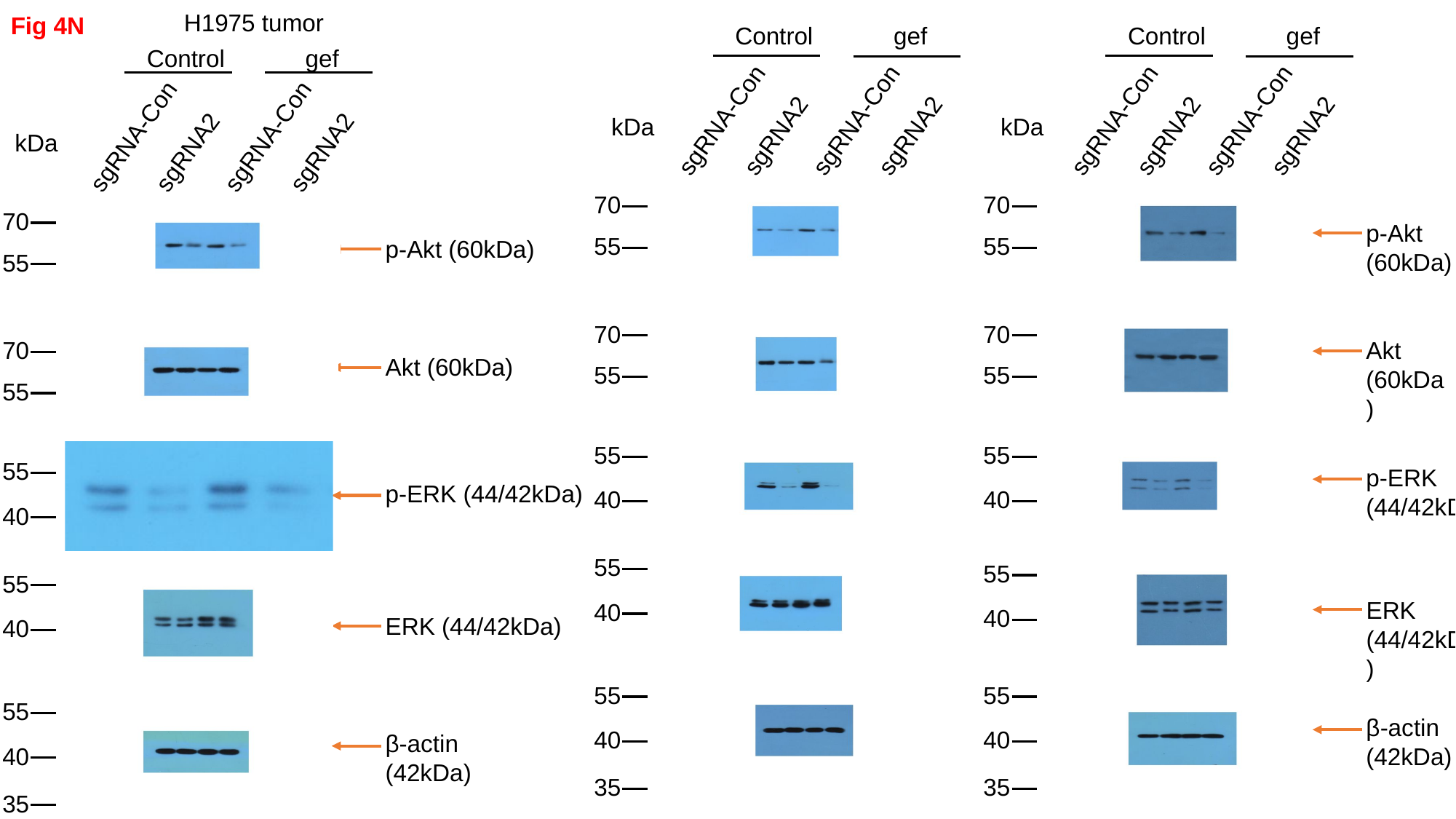

H1975 tumor
Fig 4N
Control gef
Control gef
Control gef
sgRNA-Con
sgRNA2
sgRNA-Con
sgRNA2
sgRNA-Con
sgRNA2
sgRNA-Con
sgRNA2
kDa
kDa
sgRNA-Con
sgRNA2
sgRNA-Con
sgRNA2
kDa
 70
 70
 70
p-Akt
(60kDa)
 55
 55
p-Akt (60kDa)
 55
 70
 70
 70
Akt
(60kDa)
Akt (60kDa)
 55
 55
 55
 55
 55
 55
p-ERK
(44/42kDa)
p-ERK (44/42kDa)
 40
 40
 40
 55
 55
 55
ERK
(44/42kDa)
 40
 40
ERK (44/42kDa)
 40
 55
 55
 55
β-actin
(42kDa)
 40
 40
β-actin (42kDa)
 40
 35
 35
 35

## Slide 12
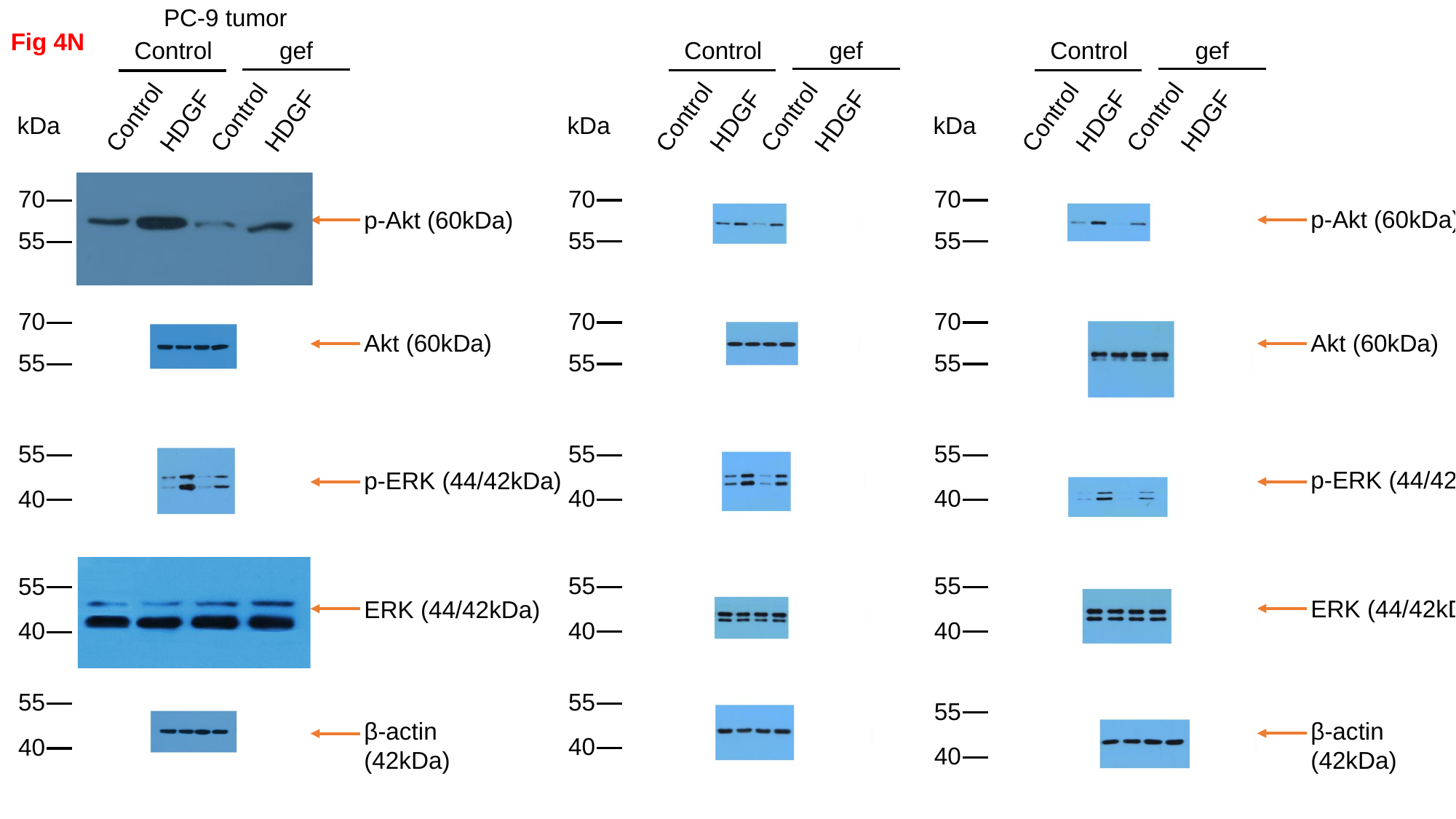

PC-9 tumor
Fig 4N
Control gef
Control gef
Control gef
Control
Control
Control
Control
Control
Control
HDGF
HDGF
HDGF
HDGF
HDGF
HDGF
kDa
kDa
kDa
 70
 70
 70
p-Akt (60kDa)
p-Akt (60kDa)
 55
 55
 55
 70
 70
 70
Akt (60kDa)
Akt (60kDa)
 55
 55
 55
 55
 55
 55
p-ERK (44/42kDa)
p-ERK (44/42kDa)
 40
 40
 40
 55
 55
 55
ERK (44/42kDa)
ERK (44/42kDa)
 40
 40
 40
 55
 55
 55
β-actin (42kDa)
β-actin (42kDa)
 40
 40
 40

## Slide 13
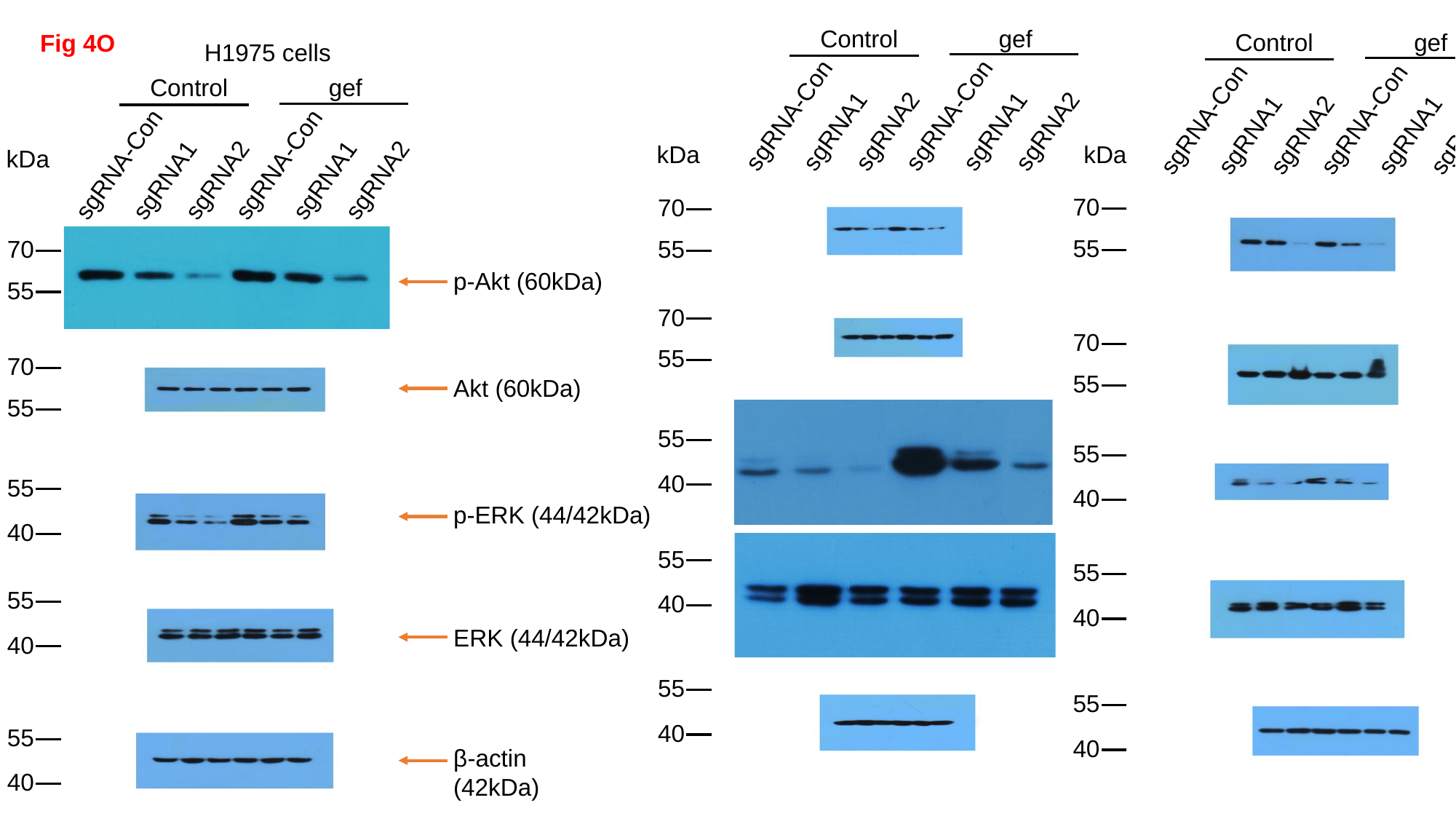

Control gef
Control gef
Fig 4O
H1975 cells
Control gef
sgRNA-Con
sgRNA1
sgRNA2
sgRNA-Con
sgRNA1
sgRNA2
sgRNA-Con
sgRNA1
sgRNA2
sgRNA-Con
sgRNA1
sgRNA2
kDa
kDa
kDa
sgRNA-Con
sgRNA1
sgRNA2
sgRNA-Con
sgRNA1
sgRNA2
 70
 70
p-Akt
(60kDa)
 55
 70
 55
p-Akt (60kDa)
 55
 70
 70
 55
 70
Akt
(60kDa)
 55
Akt (60kDa)
 55
 55
 55
p-ERK
(44/42kDa)
 40
 55
 40
p-ERK (44/42kDa)
 40
 55
 55
 55
 40
ERK
(44/42kDa)
 40
ERK (44/42kDa)
 40
 55
 55
 40
β-actin
(42kDa)
 55
 40
β-actin (42kDa)
 40

## Slide 14
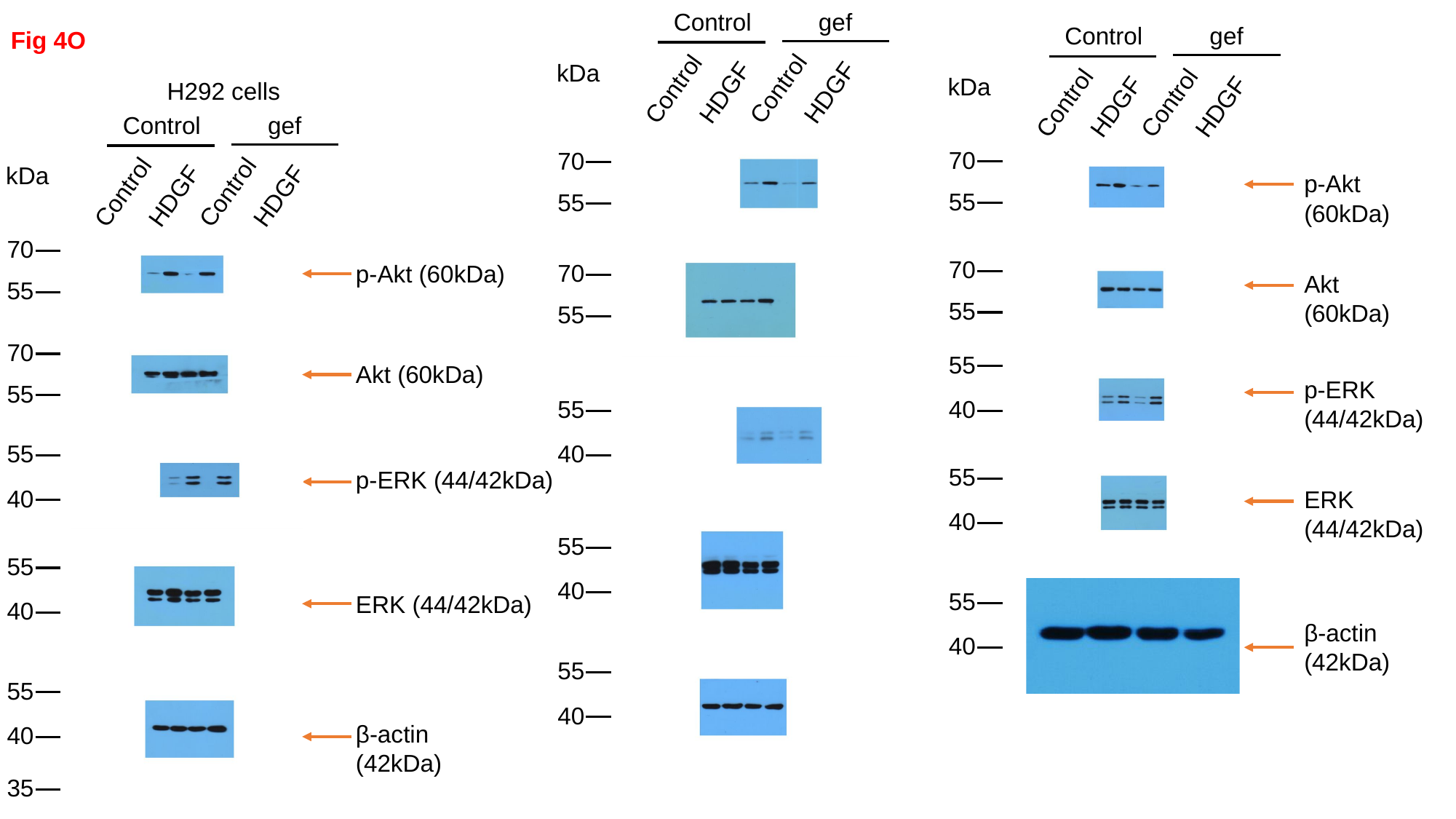

Control gef
Control gef
Fig 4O
Control
Control
kDa
Control
Control
kDa
HDGF
HDGF
H292 cells
HDGF
HDGF
Control gef
 70
 70
Control
Control
kDa
p-Akt
(60kDa)
HDGF
HDGF
 55
 55
 70
 70
 70
p-Akt (60kDa)
Akt
(60kDa)
 55
 55
 55
 70
 55
Akt (60kDa)
p-ERK
(44/42kDa)
 55
 40
 55
 55
 40
 55
p-ERK (44/42kDa)
 40
ERK
(44/42kDa)
 40
 55
 55
 40
 55
ERK (44/42kDa)
 40
β-actin
(42kDa)
 40
 55
 55
 40
β-actin (42kDa)
 40
 35

## Slide 15
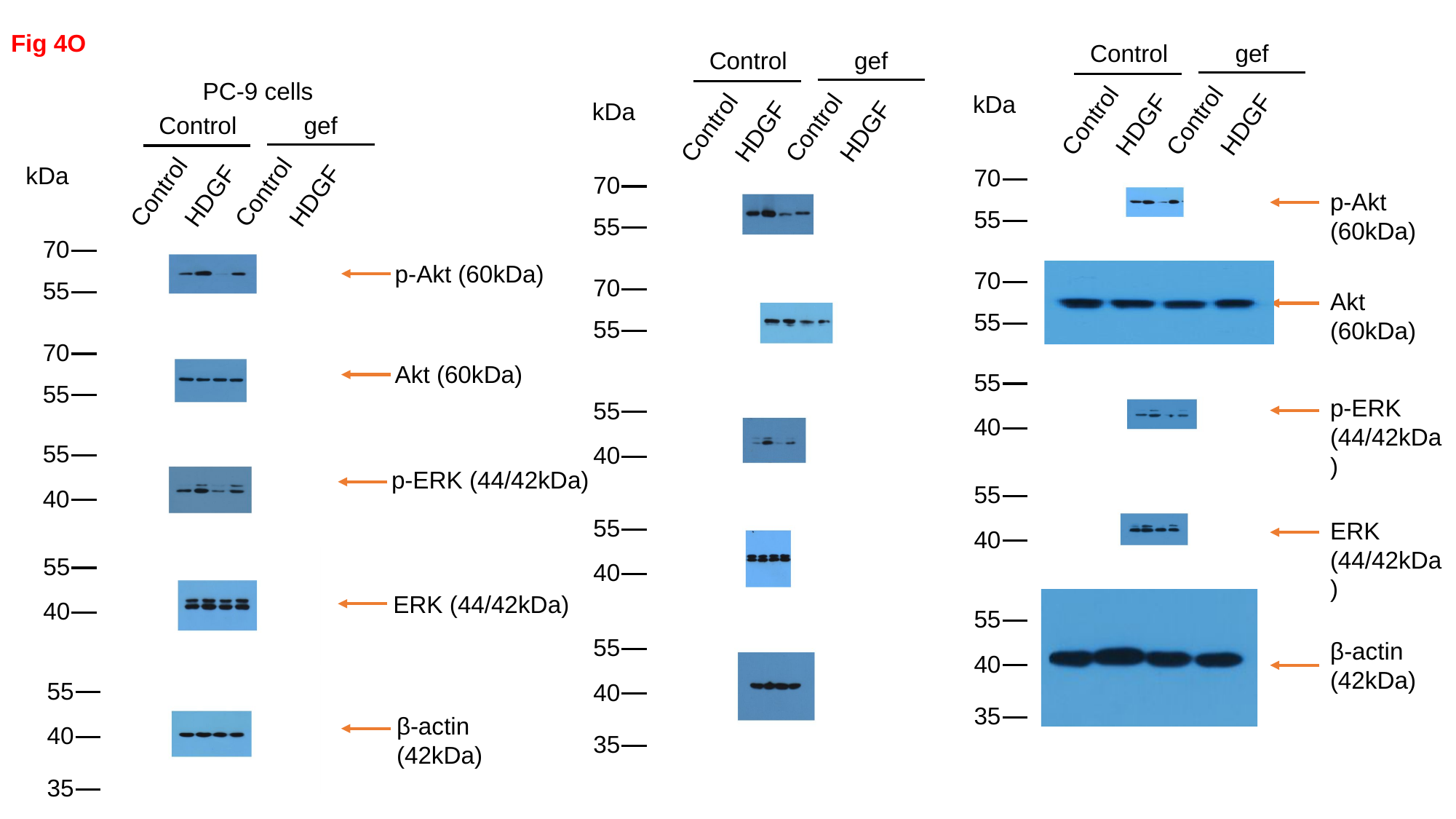

Fig 4O
Control gef
Control gef
PC-9 cells
Control
Control
kDa
Control
Control
kDa
HDGF
HDGF
Control gef
HDGF
HDGF
Control
Control
kDa
 70
 70
HDGF
HDGF
p-Akt
(60kDa)
 55
 55
 70
p-Akt (60kDa)
 70
 70
 55
Akt
(60kDa)
 55
 55
 70
Akt (60kDa)
 55
 55
p-ERK
(44/42kDa)
 55
 40
 55
 40
p-ERK (44/42kDa)
 55
 40
 55
ERK
(44/42kDa)
 40
 55
 40
ERK (44/42kDa)
 40
 55
 55
β-actin
(42kDa)
 40
 55
 40
 35
β-actin (42kDa)
 40
 35
 35

## Slide 16
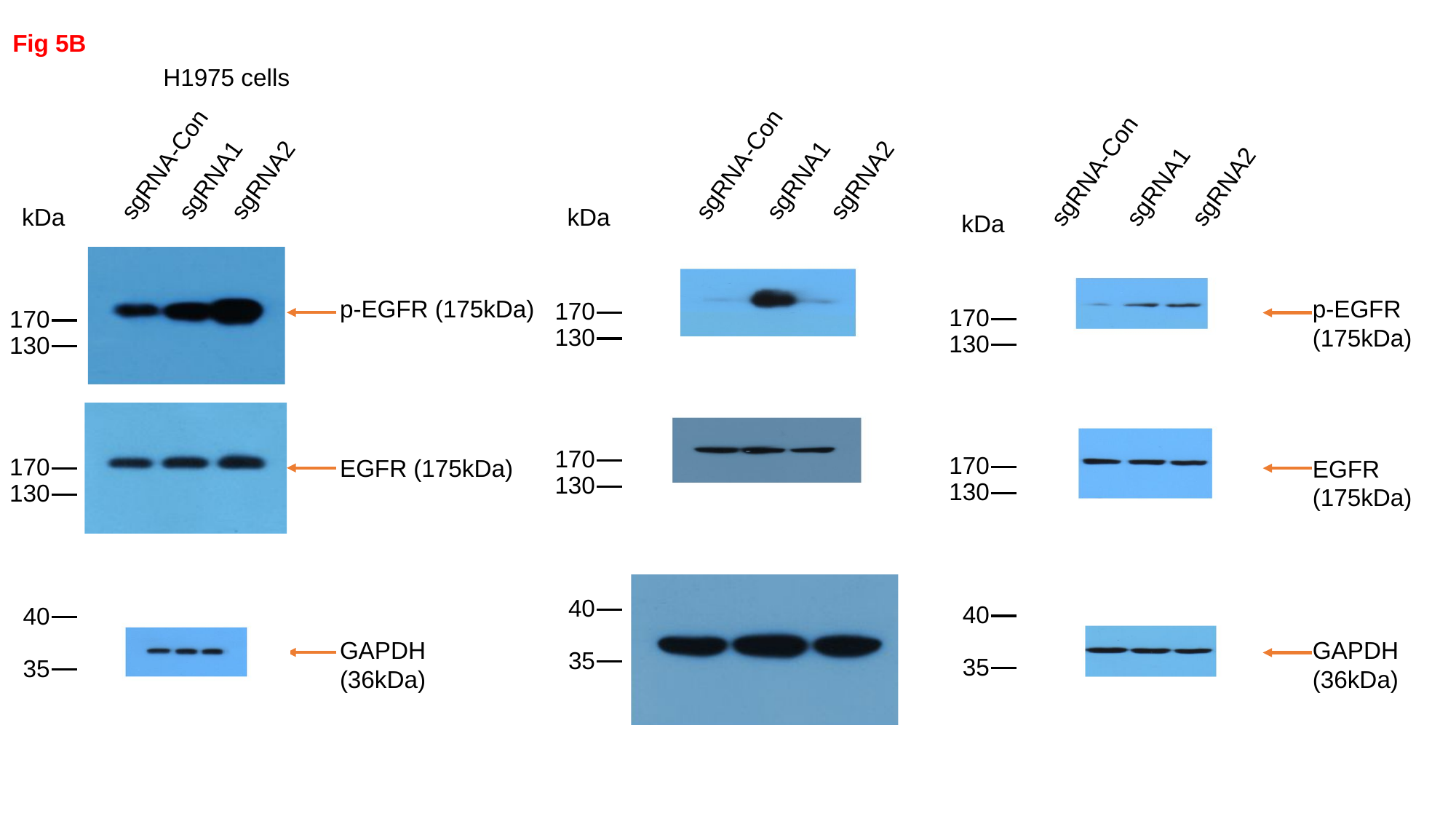

Fig 5B
H1975 cells
sgRNA-Con
sgRNA1
sgRNA2
sgRNA-Con
sgRNA1
sgRNA2
sgRNA-Con
sgRNA1
sgRNA2
kDa
kDa
kDa
p-EGFR (175kDa)
p-EGFR
(175kDa)
170
170
170
130
130
130
170
170
170
EGFR (175kDa)
EGFR
(175kDa)
130
130
130
 40
 40
 40
GAPDH (36kDa)
GAPDH
(36kDa)
 35
 35
 35

## Slide 17
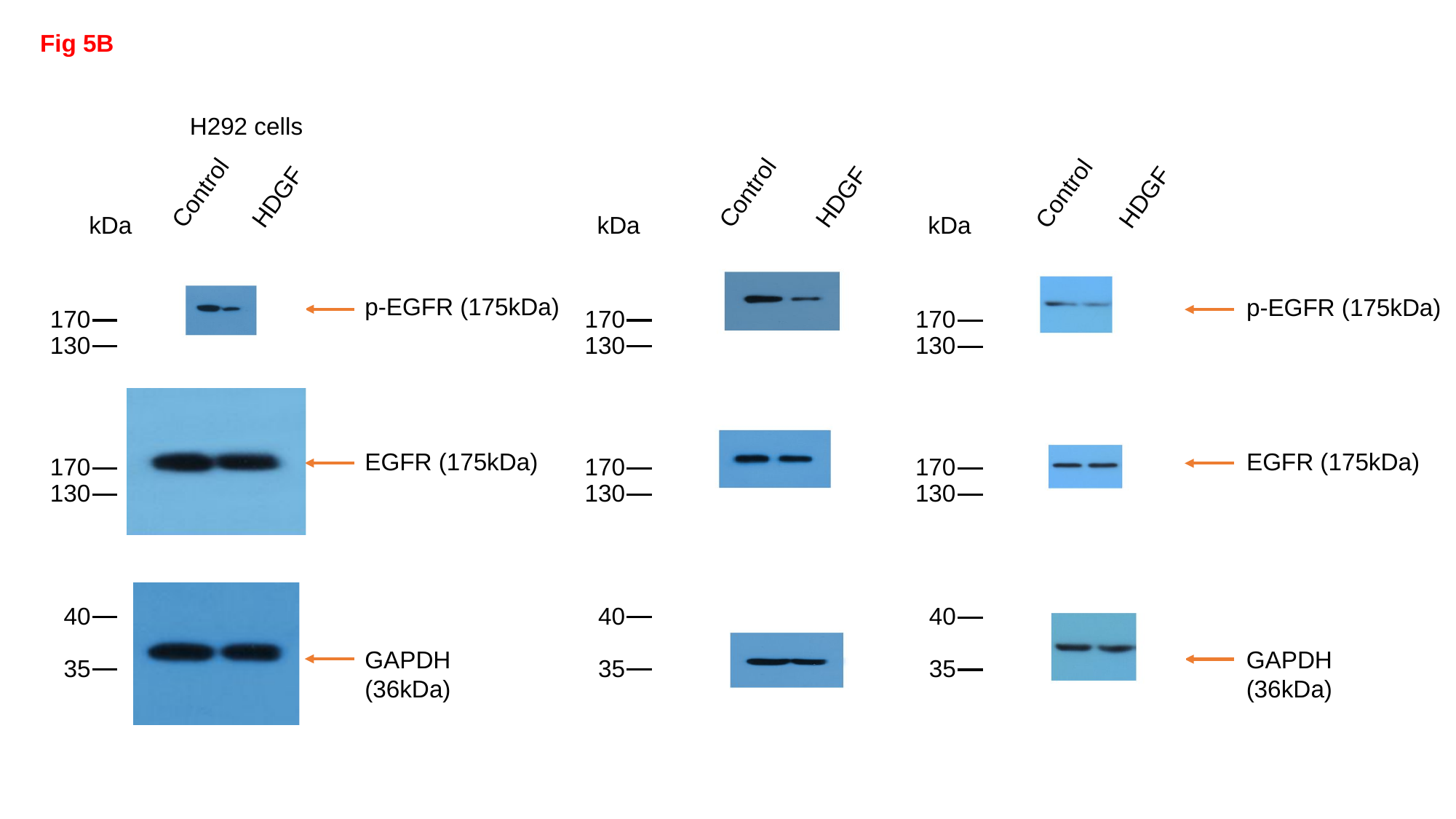

Fig 5B
H292 cells
Control
HDGF
Control
HDGF
Control
HDGF
kDa
kDa
kDa
p-EGFR (175kDa)
p-EGFR (175kDa)
170
170
170
130
130
130
EGFR (175kDa)
EGFR (175kDa)
170
170
170
130
130
130
 40
 40
 40
GAPDH (36kDa)
GAPDH (36kDa)
 35
 35
 35

## Slide 18
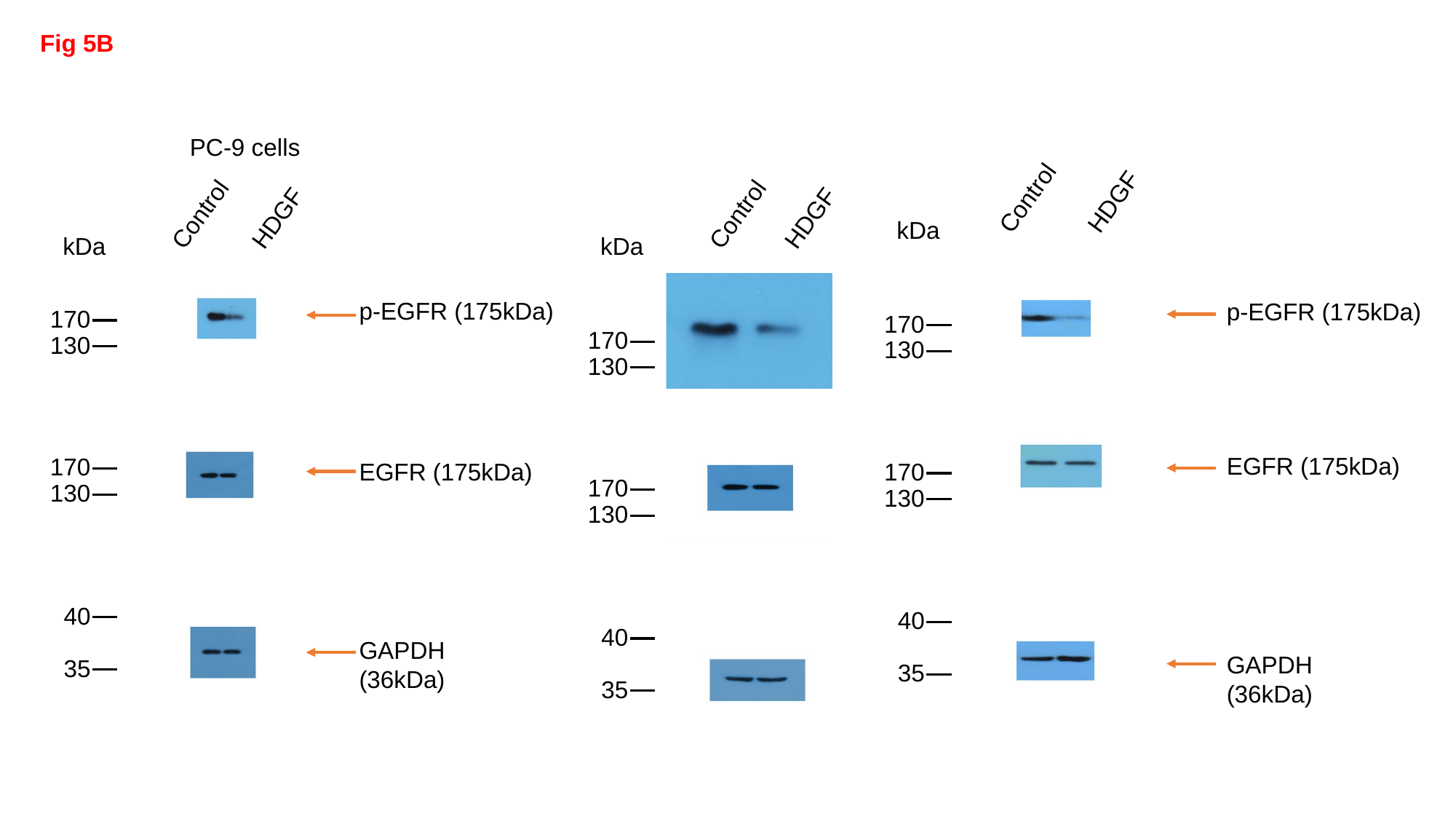

Fig 5B
PC-9 cells
Control
HDGF
Control
HDGF
Control
HDGF
kDa
kDa
kDa
p-EGFR (175kDa)
p-EGFR (175kDa)
170
170
170
130
130
130
EGFR (175kDa)
170
170
EGFR (175kDa)
170
130
130
130
 40
 40
 40
GAPDH (36kDa)
GAPDH (36kDa)
 35
 35
 35

## Slide 19
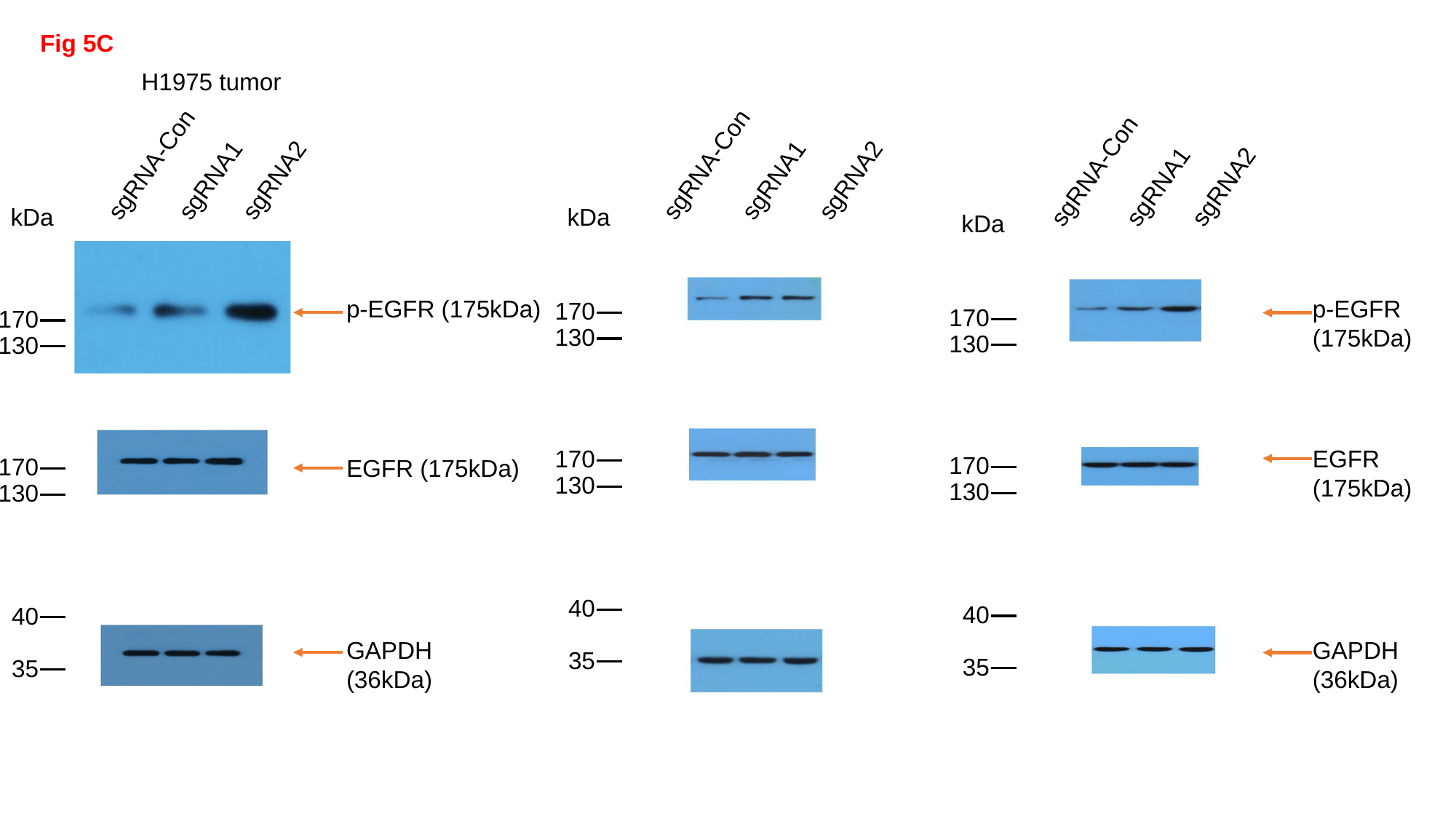

Fig 5C
H1975 tumor
sgRNA-Con
sgRNA1
sgRNA2
sgRNA-Con
sgRNA1
sgRNA2
sgRNA-Con
sgRNA1
sgRNA2
kDa
kDa
kDa
p-EGFR (175kDa)
p-EGFR
(175kDa)
170
170
170
130
130
130
EGFR
(175kDa)
170
170
170
EGFR (175kDa)
130
130
130
 40
 40
 40
GAPDH (36kDa)
GAPDH
(36kDa)
 35
 35
 35

## Slide 20
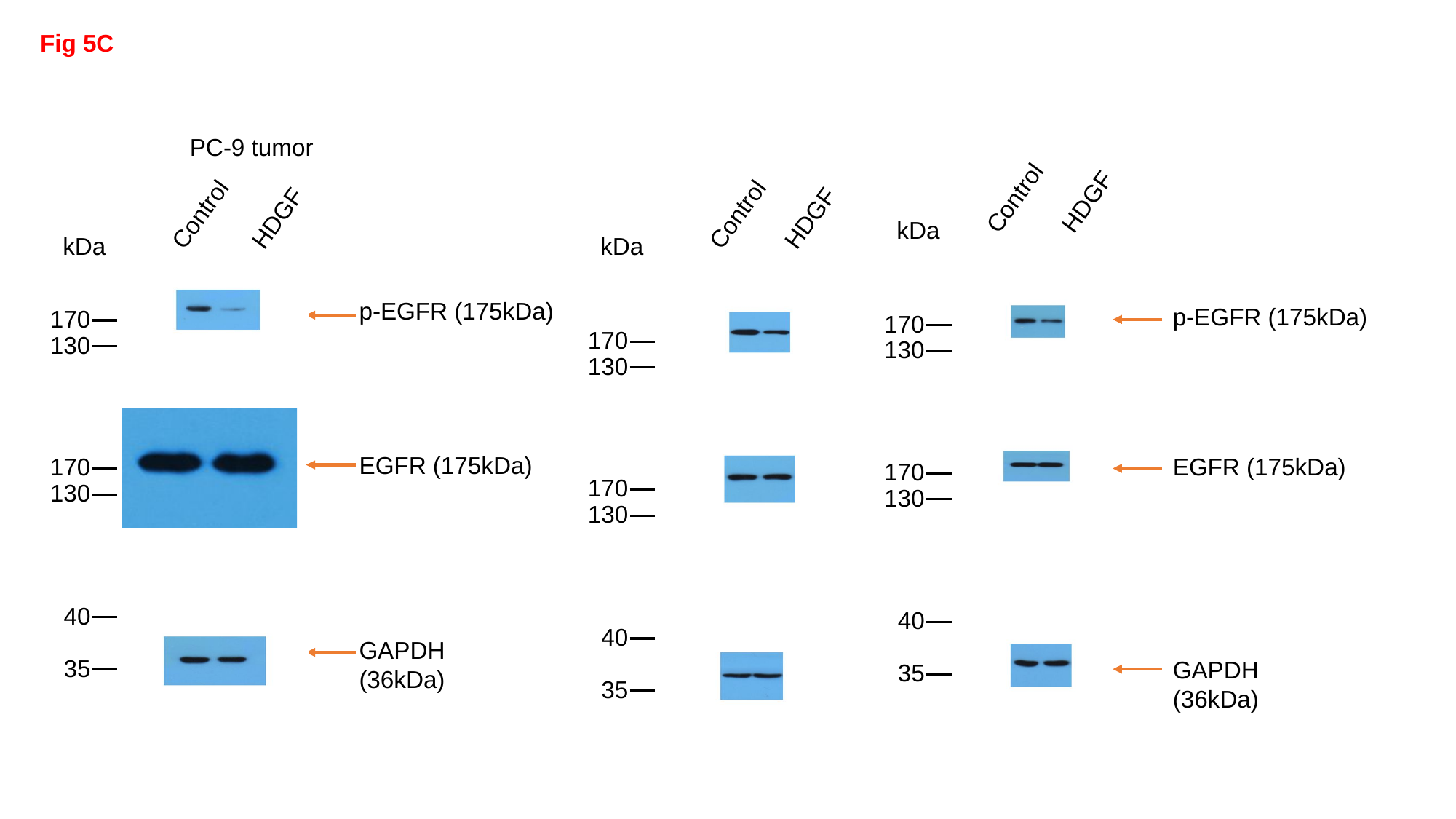

Fig 5C
PC-9 tumor
Control
HDGF
Control
HDGF
Control
HDGF
kDa
kDa
kDa
p-EGFR (175kDa)
p-EGFR (175kDa)
170
170
170
130
130
130
EGFR (175kDa)
EGFR (175kDa)
170
170
170
130
130
130
 40
 40
 40
GAPDH (36kDa)
 35
GAPDH (36kDa)
 35
 35

## Slide 21
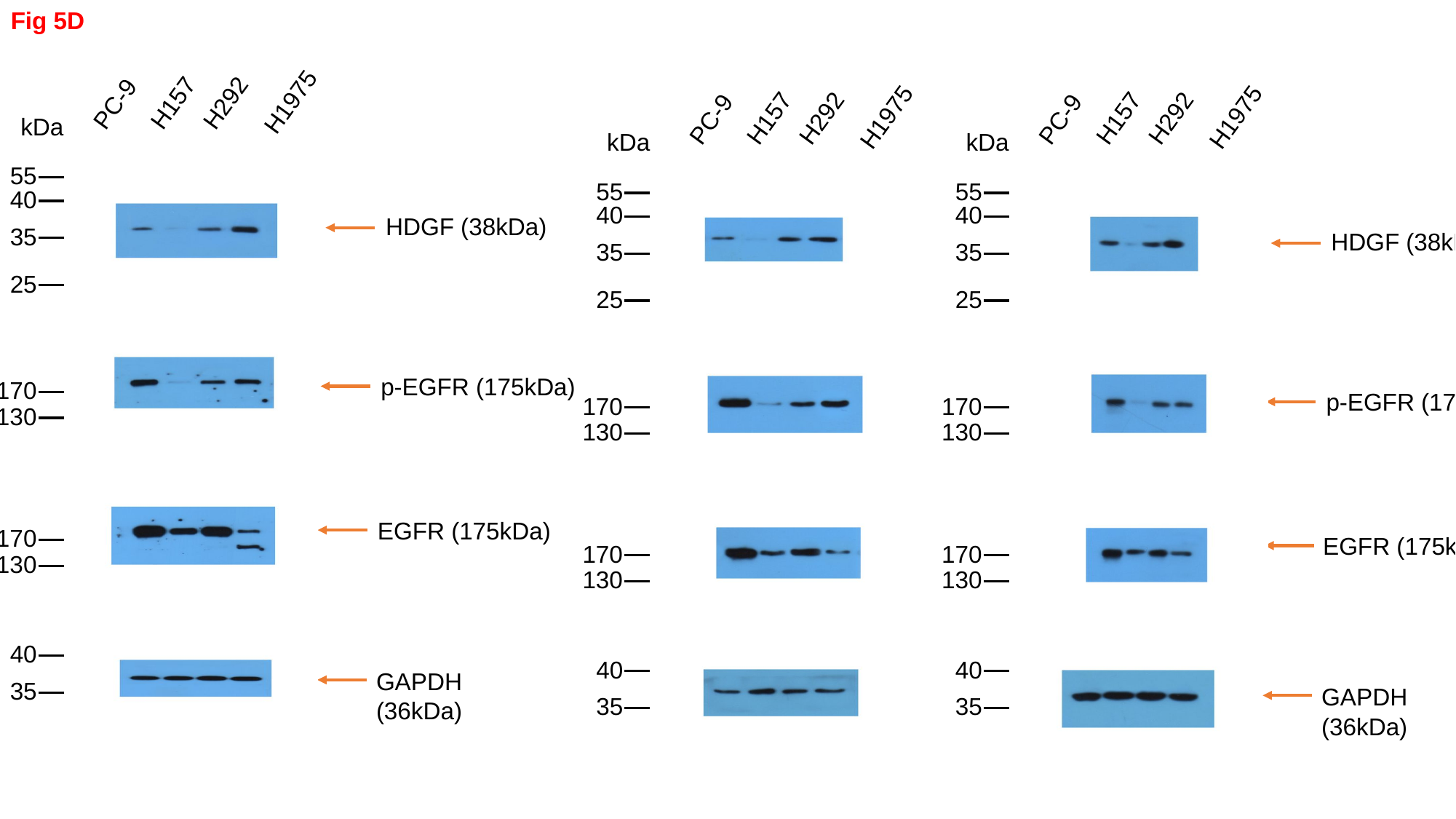

Fig 5D
PC-9
H157
H292
H1975
PC-9
H157
H292
PC-9
H157
H292
H1975
H1975
kDa
kDa
kDa
 55
 55
 55
 40
 40
 40
HDGF (38kDa)
 35
HDGF (38kDa)
 35
 35
 25
 25
 25
p-EGFR (175kDa)
170
p-EGFR (175kDa)
170
170
130
130
130
EGFR (175kDa)
170
EGFR (175kDa)
170
170
130
130
130
 40
 40
 40
GAPDH (36kDa)
 35
GAPDH (36kDa)
 35
 35

## Slide 22
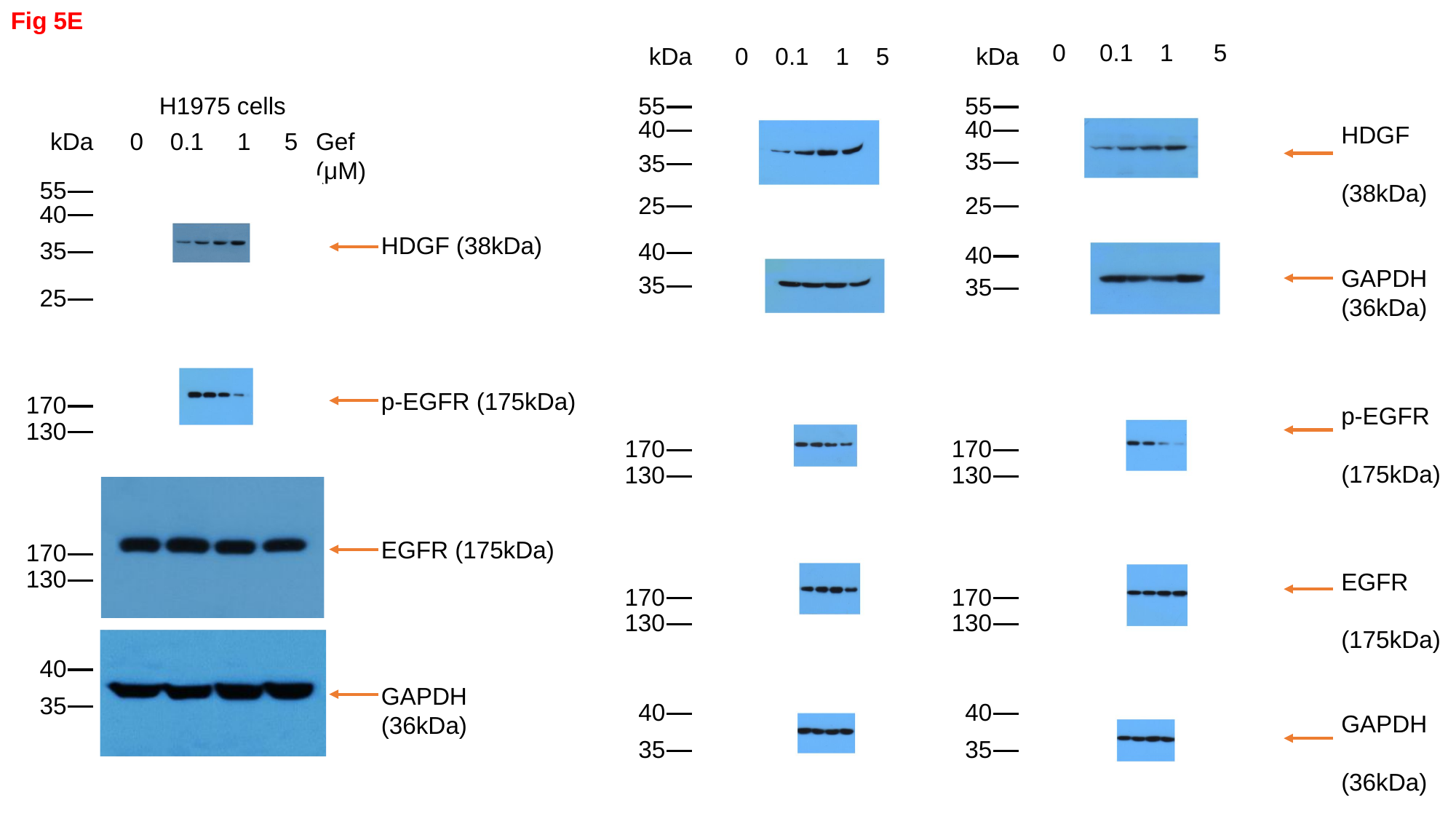

Fig 5E
0 0.1 1 5
kDa
0 0.1 1 5
kDa
H1975 cells
 55
 55
 40
 40
HDGF
 (38kDa)
kDa
0 0.1 1 5
Gef (μM)
 35
 35
 55
 25
 25
 40
HDGF (38kDa)
 35
 40
 40
GAPDH
(36kDa)
 35
 35
 25
p-EGFR (175kDa)
170
p-EGFR
 (175kDa)
130
170
170
130
130
EGFR (175kDa)
170
130
EGFR
 (175kDa)
170
170
130
130
 40
GAPDH (36kDa)
 35
 40
 40
GAPDH
 (36kDa)
 35
 35

## Slide 23
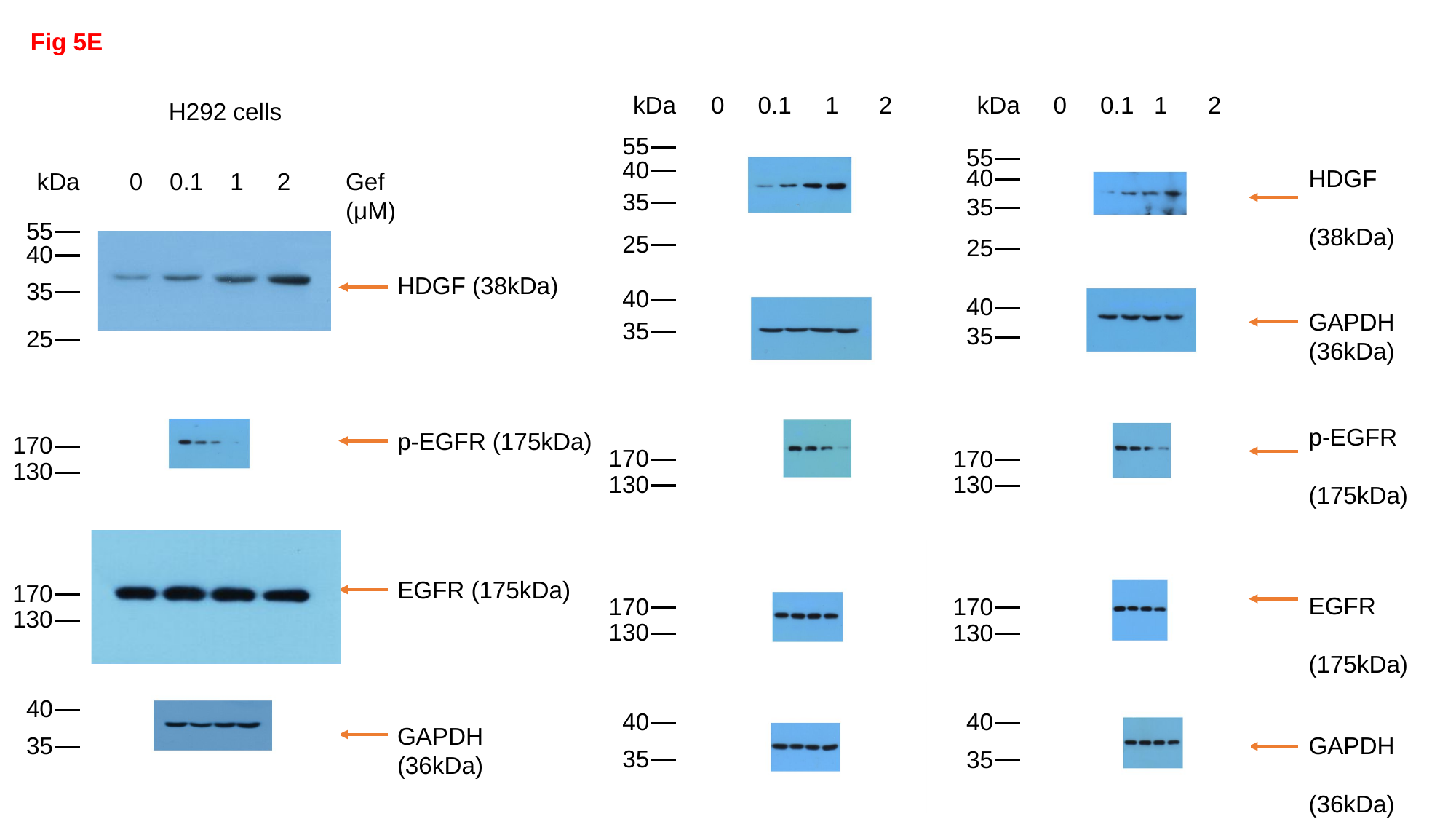

Fig 5E
kDa
0 0.1 1 2
kDa
0 0.1 1 2
H292 cells
 55
 55
 40
 40
HDGF
 (38kDa)
kDa
0 0.1 1 2
Gef (μM)
 35
 35
 55
 25
 25
 40
HDGF (38kDa)
 35
 40
 40
GAPDH
(36kDa)
 35
 35
 25
p-EGFR
 (175kDa)
p-EGFR (175kDa)
170
170
170
130
130
130
EGFR (175kDa)
170
EGFR
 (175kDa)
170
170
130
130
130
 40
 40
 40
GAPDH (36kDa)
GAPDH
 (36kDa)
 35
 35
 35

## Slide 24
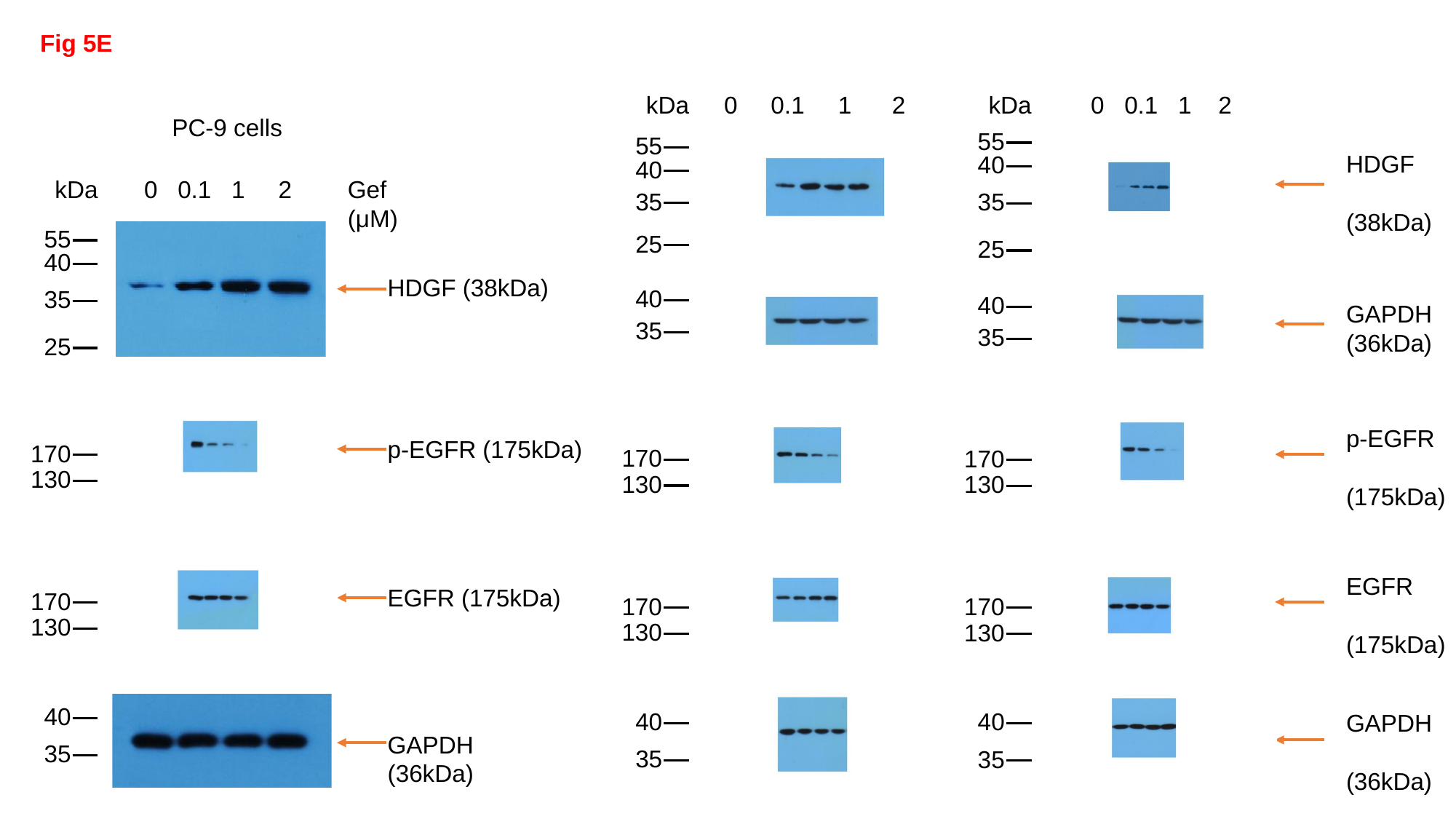

Fig 5E
kDa
0 0.1 1 2
kDa
0 0.1 1 2
PC-9 cells
 55
 55
HDGF
 (38kDa)
 40
 40
kDa
0 0.1 1 2
Gef (μM)
 35
 35
 55
 25
 25
 40
HDGF (38kDa)
 40
 35
 40
GAPDH
(36kDa)
 35
 35
 25
p-EGFR
 (175kDa)
p-EGFR (175kDa)
170
170
170
130
130
130
EGFR
 (175kDa)
EGFR (175kDa)
170
170
170
130
130
130
 40
 40
 40
GAPDH
 (36kDa)
GAPDH (36kDa)
 35
 35
 35

## Slide 25
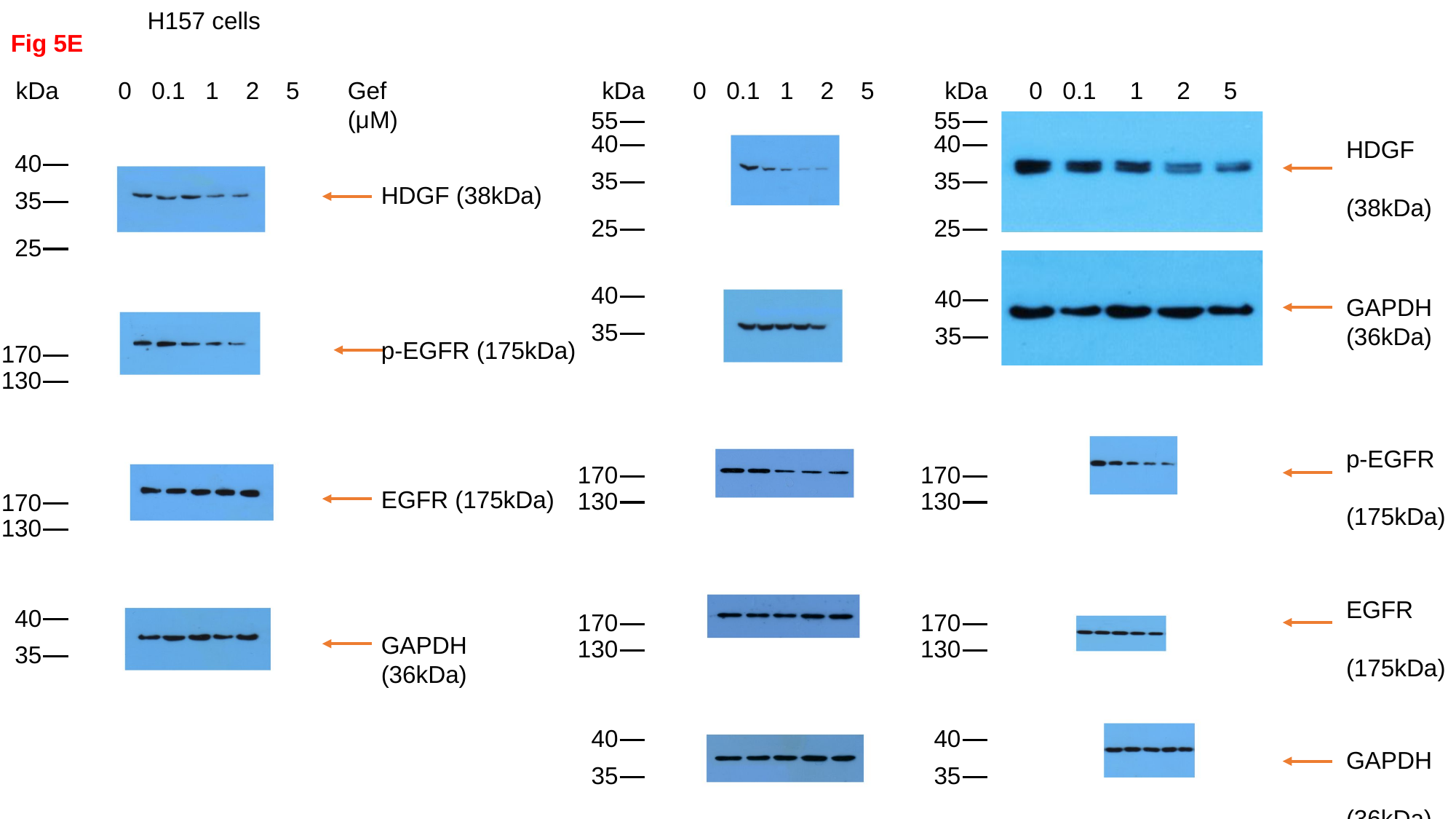

H157 cells
Fig 5E
kDa
0 0.1 1 2 5
Gef (μM)
kDa
0 0.1 1 2 5
kDa
0 0.1 1 2 5
 55
 55
 40
 40
HDGF
 (38kDa)
 40
 35
 35
HDGF (38kDa)
 35
 25
 25
 55
 25
 40
 40
GAPDH
(36kDa)
 35
 35
p-EGFR (175kDa)
170
130
p-EGFR
 (175kDa)
170
170
EGFR (175kDa)
130
130
170
130
EGFR
 (175kDa)
 40
170
170
GAPDH (36kDa)
130
130
 35
 40
 40
GAPDH
 (36kDa)
 35
 35

## Slide 26
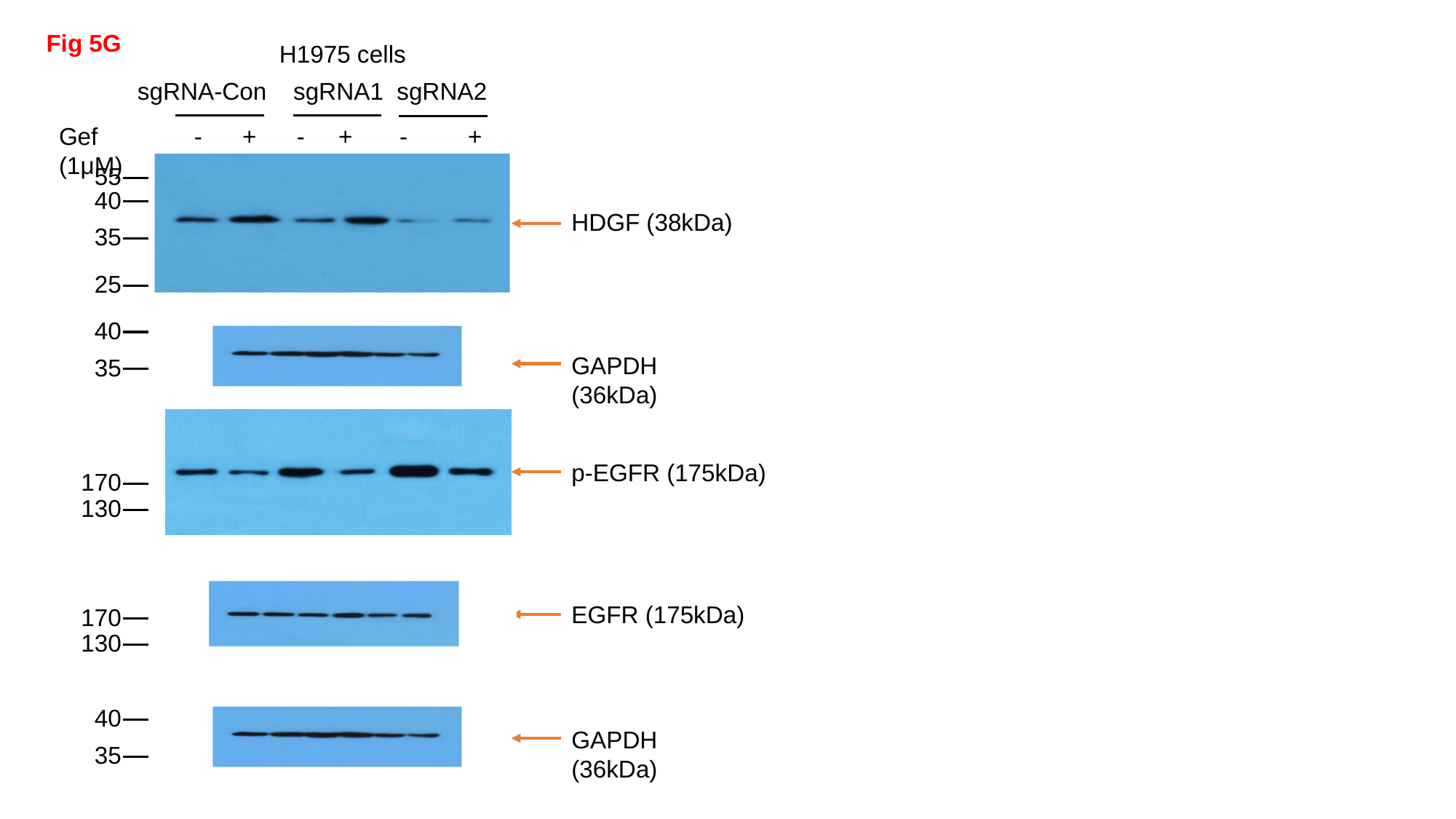

Fig 5G
H1975 cells
sgRNA-Con sgRNA1 sgRNA2
Gef (1μM)
- + - + - +
 55
 40
HDGF (38kDa)
 35
 25
 40
GAPDH (36kDa)
 35
p-EGFR (175kDa)
170
130
EGFR (175kDa)
170
130
 40
GAPDH (36kDa)
 35

## Slide 27
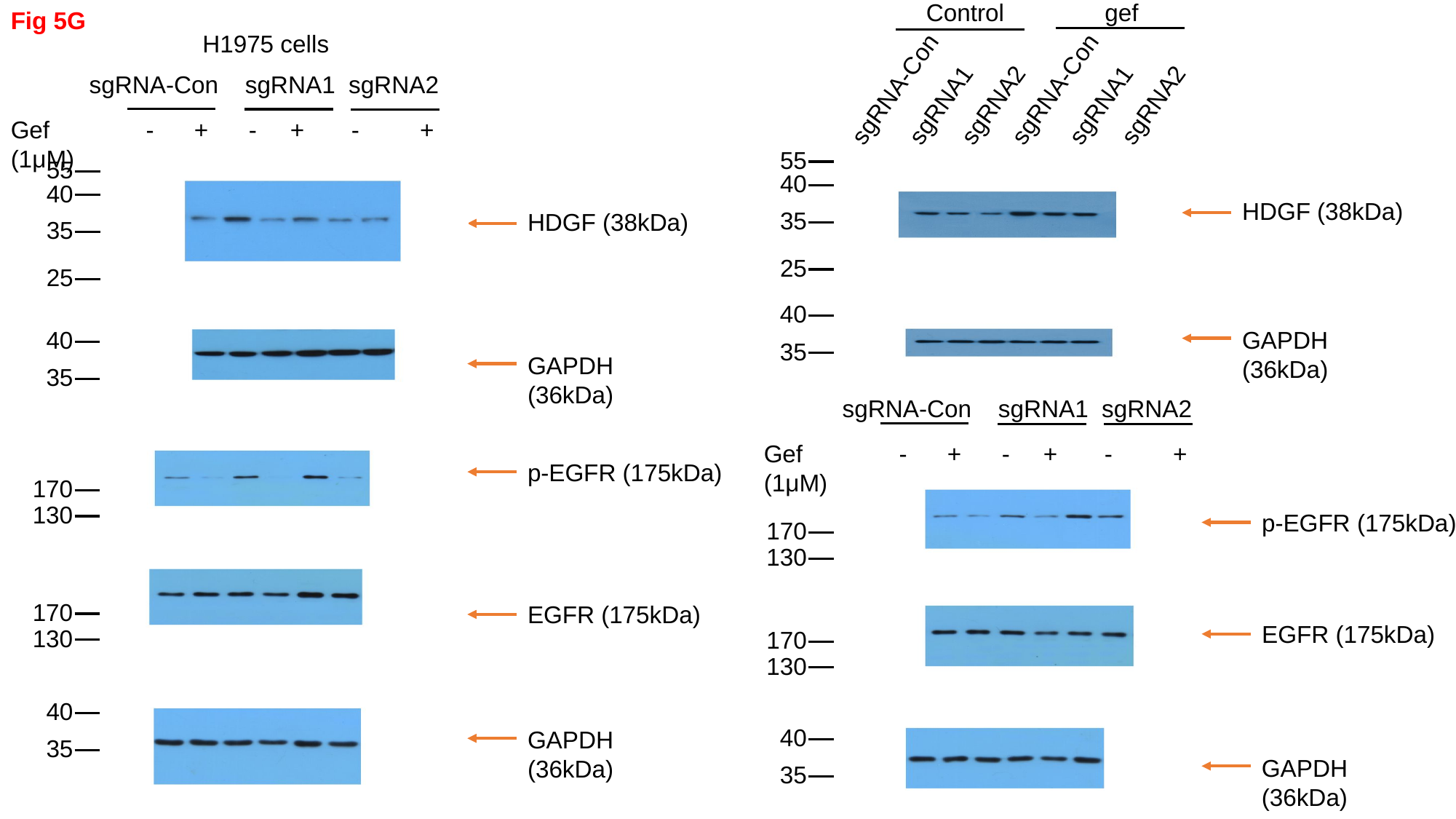

Fig 5G
Control gef
H1975 cells
sgRNA-Con sgRNA1 sgRNA2
sgRNA-Con
sgRNA1
sgRNA2
sgRNA-Con
sgRNA1
sgRNA2
Gef (1μM)
- + - + - +
 55
 55
 40
 40
HDGF (38kDa)
 35
HDGF (38kDa)
 35
 25
 25
 40
GAPDH (36kDa)
 40
 35
GAPDH (36kDa)
 35
sgRNA-Con sgRNA1 sgRNA2
Gef (1μM)
- + - + - +
p-EGFR (175kDa)
170
130
p-EGFR (175kDa)
170
130
170
EGFR (175kDa)
EGFR (175kDa)
130
170
130
 40
 40
GAPDH (36kDa)
 35
GAPDH (36kDa)
 35

## Slide 28
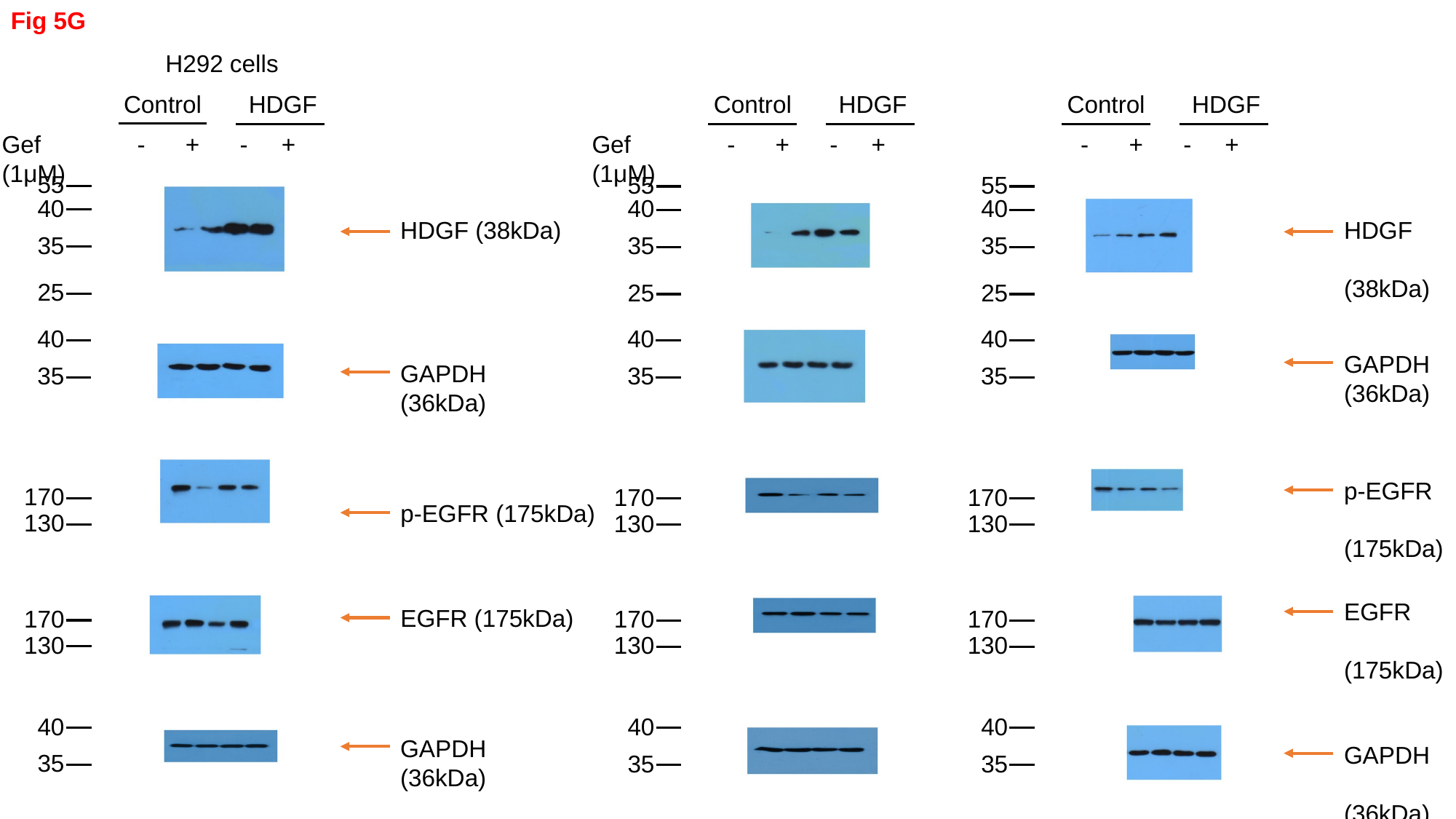

Fig 5G
H292 cells
Control HDGF
Control HDGF
Control HDGF
Gef (1μM)
- + - +
Gef (1μM)
- + - +
- + - +
 55
 55
 55
 40
 40
 40
HDGF (38kDa)
HDGF
 (38kDa)
 35
 35
 35
 25
 25
 25
 40
 40
 40
GAPDH
(36kDa)
GAPDH (36kDa)
 35
 35
 35
p-EGFR
 (175kDa)
170
170
170
p-EGFR (175kDa)
130
130
130
EGFR
 (175kDa)
EGFR (175kDa)
170
170
170
130
130
130
 40
 40
 40
GAPDH (36kDa)
GAPDH
 (36kDa)
 35
 35
 35

## Slide 29
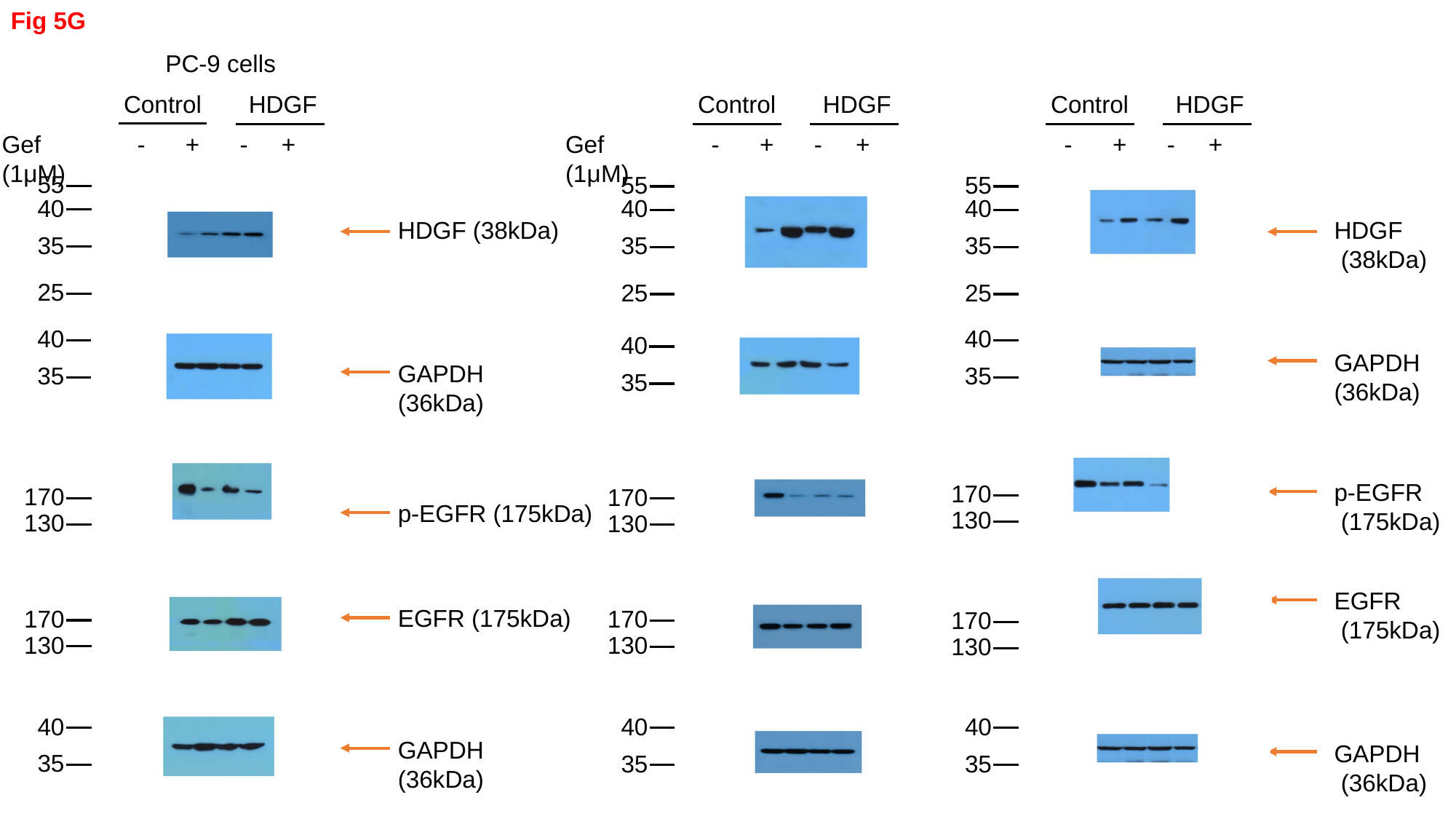

Fig 5G
PC-9 cells
Control HDGF
Control HDGF
Control HDGF
Gef (1μM)
- + - +
Gef (1μM)
- + - +
- + - +
 55
 55
 55
 40
 40
 40
HDGF (38kDa)
HDGF
 (38kDa)
 35
 35
 35
 25
 25
 25
 40
 40
 40
GAPDH
(36kDa)
GAPDH (36kDa)
 35
 35
 35
p-EGFR
 (175kDa)
170
170
170
p-EGFR (175kDa)
130
130
130
EGFR
 (175kDa)
EGFR (175kDa)
170
170
170
130
130
130
 40
 40
 40
GAPDH (36kDa)
GAPDH
 (36kDa)
 35
 35
 35

## Slide 30
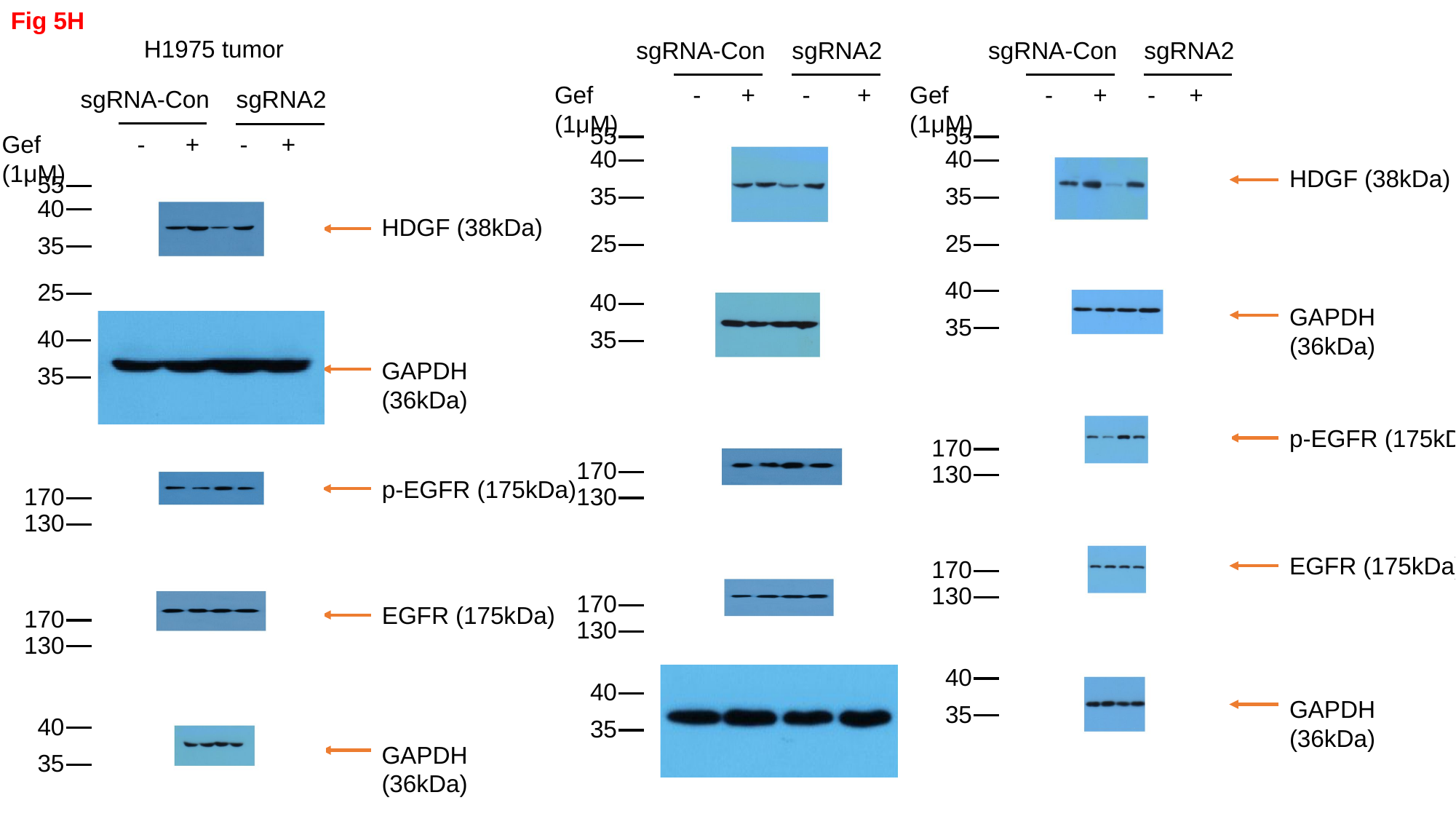

Fig 5H
H1975 tumor
sgRNA-Con sgRNA2
sgRNA-Con sgRNA2
Gef (1μM)
- + - +
Gef (1μM)
- + - +
sgRNA-Con sgRNA2
 55
 55
Gef (1μM)
- + - +
 40
 40
HDGF (38kDa)
 55
 35
 35
 40
HDGF (38kDa)
 25
 25
 35
 40
 25
 40
GAPDH (36kDa)
 35
 40
 35
GAPDH (36kDa)
 35
p-EGFR (175kDa)
170
170
130
p-EGFR (175kDa)
130
170
130
EGFR (175kDa)
170
130
170
EGFR (175kDa)
170
130
130
 40
 40
GAPDH (36kDa)
 35
 40
 35
GAPDH (36kDa)
 35

## Slide 31
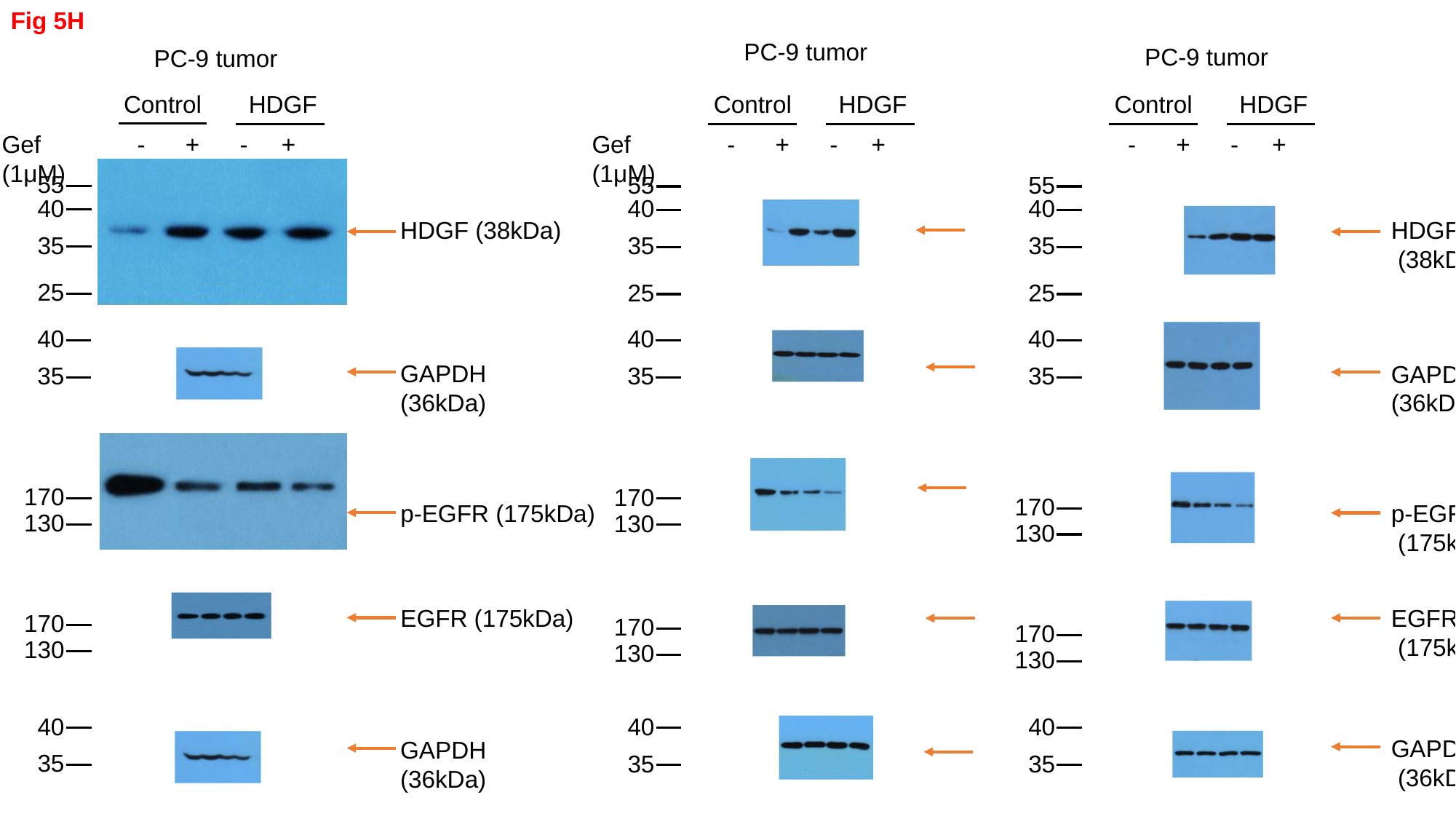

Fig 5H
PC-9 tumor
PC-9 tumor
PC-9 tumor
Control HDGF
Control HDGF
Control HDGF
Gef (1μM)
- + - +
Gef (1μM)
- + - +
- + - +
 55
 55
 55
 40
 40
 40
HDGF (38kDa)
HDGF
 (38kDa)
 35
 35
 35
 25
 25
 25
 40
 40
 40
GAPDH (36kDa)
GAPDH
(36kDa)
 35
 35
 35
170
170
170
p-EGFR (175kDa)
p-EGFR
 (175kDa)
130
130
130
EGFR (175kDa)
EGFR
 (175kDa)
170
170
170
130
130
130
 40
 40
 40
GAPDH
 (36kDa)
GAPDH (36kDa)
 35
 35
 35

## Slide 32
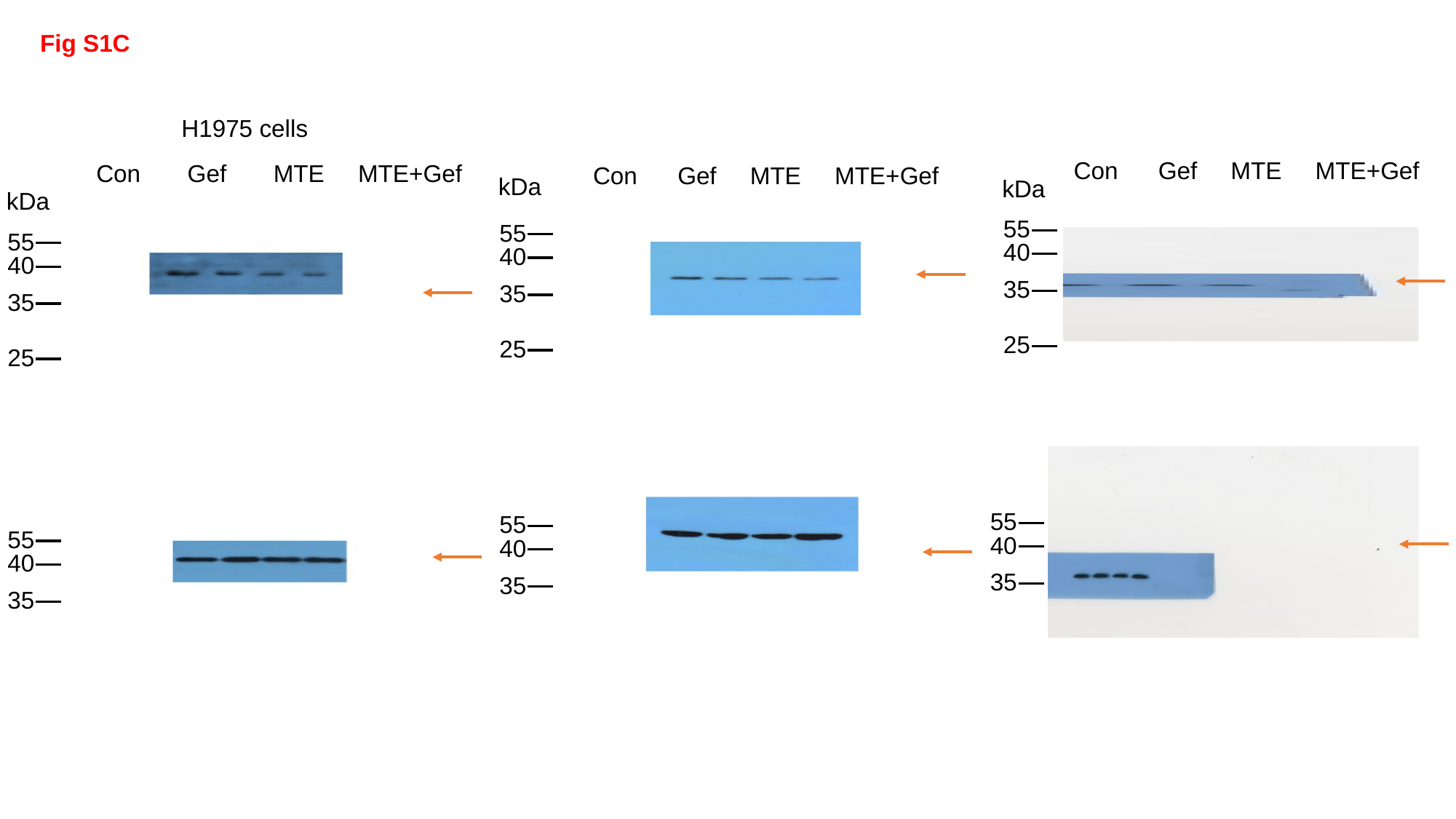

Fig S1C
H1975 cells
Con Gef MTE MTE+Gef
Con Gef MTE MTE+Gef
Con Gef MTE MTE+Gef
kDa
kDa
kDa
 55
 55
 55
 40
 40
 40
HDGF
 (38kDa)
 35
 35
 35
 25
 25
 25
 55
 55
 55
β-actin
(42kDa)
 40
 40
 40
 35
 35
 35

## Slide 33
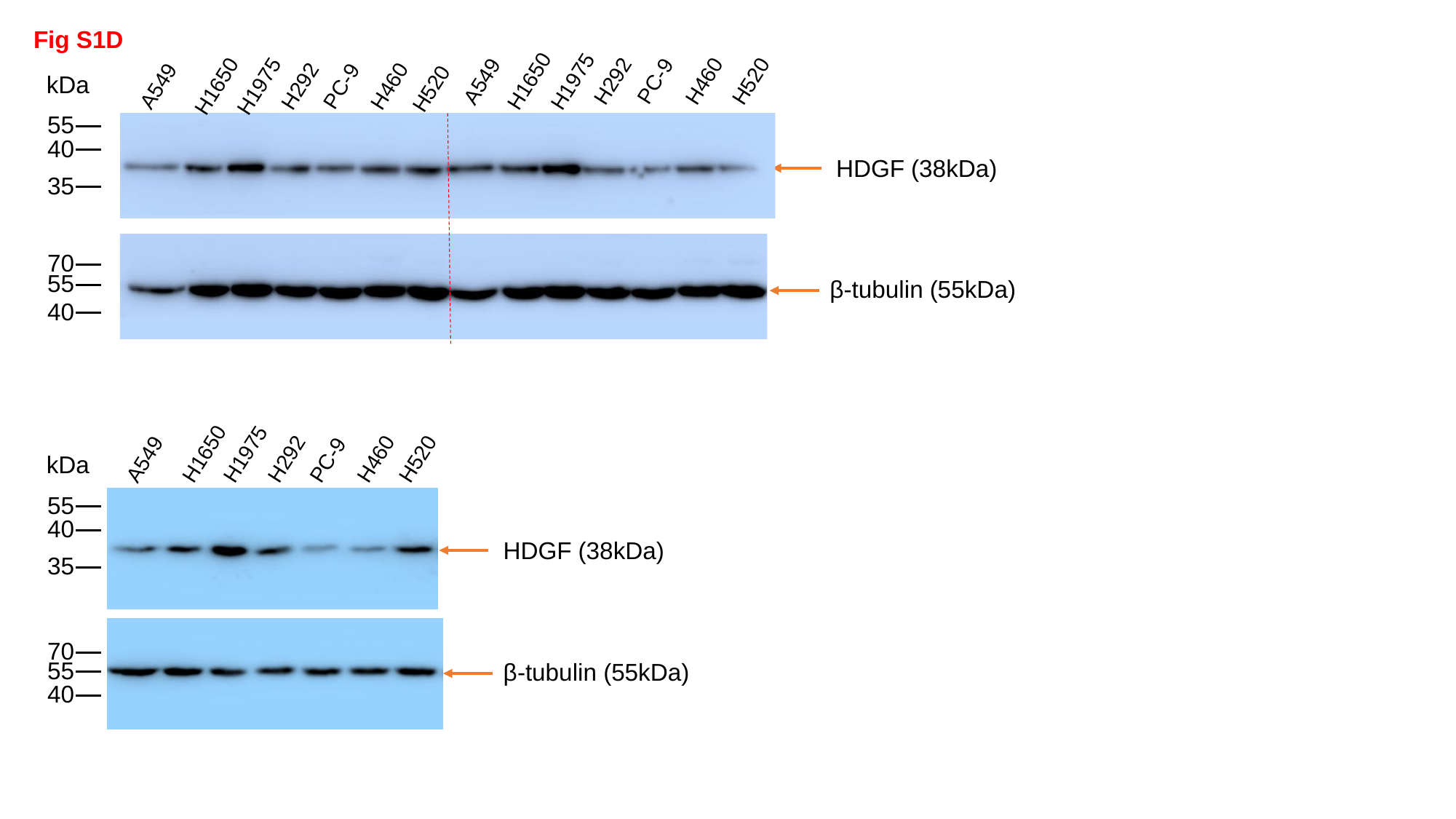

Fig S1D
A549
H1650
H1975
H292
PC-9
H460
H520
A549
H1650
H1975
H292
PC-9
H460
H520
kDa
 55
 40
HDGF (38kDa)
 35
 70
 55
β-tubulin (55kDa)
 40
H1650
H1975
H292
H460
H520
A549
PC-9
kDa
 55
 40
HDGF (38kDa)
 35
 70
 55
β-tubulin (55kDa)
 40
